# Genetic characteristics and ploidy trigger the high inducibility of double haploid (DH) inducer in *Brassica napus*

**DOI:** 10.1186/s12870-021-03311-z

**Published:** 2021-11-16

**Authors:** Xuan Luo, Jin Yang, Zhendong Zhu, Liangjun Huang, Asif Ali, Hafiz Hassan Javed, Wei Zhang, Ying Zhou, Liqin Yin, Peizhou Xu, Xingyu Liang, Yun Li, Jisheng Wang, Qiong Zou, Wanzhuo Gong, Haoran Shi, Lanrong Tao, Zeming Kang, Rong Tang, Hailan Liu, Shaohong Fu

**Affiliations:** 1 Maize Research Institute, Sichuan Agricultural University, Chengdu, 611130 China; 2 Institute of Crop Research, Chengdu Academy of Agricultural and Forestry Sciences, Chengdu, 611130 China; 3 Chengdu Research Branch, National Rapeseed Genetic Improvement Center, Chengdu, 611130 China; 4 Agricultural College, Sichuan Agricultural University, Chengdu, 611130 China; 5 Rice Research Institute, Sichuan Agricultural University, Chengdu, 611130 China

**Keywords:** Doubled haploid inducer, Flow cytometry, Phenotypic identification, SNP genotyping, Homozygous sites rate, Aneuploidy, Ploidy, *Bassica napus* L

## Abstract

**Background:**

Our recently reported doubled haploid (DH) induction lines e.g., Y3380 and Y3560 are allo-octoploid (AAAACCCC, 2n = 8× ≈ 76), which can induce the maternal parent to produce DH individuals. Whether this induction process is related to the production of aneuploid gametes form male parent and genetic characteristics of the male parent has not been reported yet.

**Results:**

Somatic chromosome counts of DH inducer parents, female wax-less parent (W1A) and their F_1_ hybrid individuals revealed the reliability of flow cytometry analysis. Y3560 has normal chromosome behavior in metaphase I and anaphase I, but chromosome division was not synchronized in the tetrad period. Individual phenotypic identification and flow cytometric fluorescence measurement of F_1_ individual and parents revealed that DH individuals can be distinguished on the basis of waxiness trait. The results of phenotypic identification and flow cytometry can identify the homozygotes or heterozygotes of F_1_ generation individuals. The data of SNP genotyping coupled with phenotypic waxiness trait revealed that the genetic distance between W1A and F_1_ homozygotes were smaller as compared to their heterozygotes. It was found that compared with allo-octoploids, aneuploidy from allo-octoploid segregation did not significantly increase the DH induction rate, but reduced male infiltration rate and heterozygous site rate of induced F_1_ generation. The ploidy, SNP genotyping and flow cytometry results cumulatively shows that DH induction is attributed to the key genes regulation from the parents of Y3560 and Y3380, which significantly increase the induction efficiency as compared to ploidy.

**Conclusion:**

Based on our findings, we hypothesize that genetic characteristics and aneuploidy play an important role in the induction of DH individuals in *Brassca napus*, and the induction process has been explored. It provides an important insight for us to locate and clone the genes that regulate the inducibility in the later stage.

**Supplementary Information:**

The online version contains supplementary material available at 10.1186/s12870-021-03311-z.

## Background

*Brassica* species, with phenomenon of genome duplication and gene combination [1] have been used as a polyploid genome model for studying plant evolution. *Brassica napus* is one of the earliest allopolyploid crops, which originated from the Mediterranean region about 7500 years ago. It is naturally occurring hybrid and its doubling is formed with *B. rapa* (AA, 2n = 20) and *B. oleracea* (CC, 2n = 18) [1, 2]. *Brassica napus* (AACC, 2n = 4× = 38) is an allo-tetraploid and breeders face difficulty to produce pure lines, which usually takes 6 ~ 8 years to breed a pure line. Microspore in vitro culture technology is being used to obtain haploids and double haploids (DH) in early generations [3]. However, micro-spore culture requires a high experimental expertise that makes it costly and cumbersome [4]. The elimination of single parental chromosome has been observed to produce haploids in maize [5], wheat [6], barley [7], potato [8], and arabidopsis [9]. Afterwards, DH lines are obtained by artificial doubling of haploid chromosome. The induction mechanism of DH line has been studied in maize that revealed exclusion of chromosome [10]. Key inducing genes *ZmDMP* [11] and *ZmPLA1* [12] are important player in DH induction systems in many species. In our previous study we bred Y3380 and Y3560, allo-octoploids (2n = 8× ≈ 76, AAAACCCC) rapeseed through interspecific hybridization and genome doubling [13]. Another of our report presents that when these allo-octoploid were used as pollen donors, the offspring are DH plants [14]. The pollen of Y3380 was used to mediate gene editing in *Brassica napus* and *Brassica oleracea* to produce female double haploid progenies without editing vector sequence [15]. In the offspring, most of the genome was inherited from the maternal parent, with a smaller infiltration from the male parent, and their induction efficiency ranges from 34.09 ~ 98.66%. That’s why Y3380 and Y3560 were named as allo-octoploid DH inducer in *Brassica napus* [13, 14]. These DH inducers are advantageous, as artificial doubling of haploid chromosome is not needed, and can directly induce the DH lines in a single step [13–15]. This novel method provides new insights to study further generation of homozygous lines, moreover, it speeds up the selection and breeding of rapeseed varieties. There are only a few studies that focus on the induction mechanism of DH in *Brassica napus.* In order to accelerate rapeseed breeding, exploring the induction mechanism of DH inducer will provide a theoretical basis for further innovation of germplasm resources. In maize, single-cell sequencing revealed that haploid induction is mainly caused by the production of aneuploid gametes from the pollen of the inducer [16]. The meiosis of artificially synthesized allo-octoploid Y3380 and Y3560 has presented obvious abnormalities, and ploidy of their self-progeny has also segregation phenotypes [17]. Therefore, the inbred progenies of Y3380 and Y3560 showed a normal distribution of ploidy segregation, including tetraploid, hexaploid, octoploid, decaploid, and dodecaploid accounted for about 17 ~ 40%, and aneuploid or mixed-ploid were also isolated [17]. Did the maternal haploids or DH have been obtained from the aneuploid pollen of the inducer male parent? In addition, do other polyploids (hexaploid and octoploid) have ability of induction? Does the ability of induction come from aneuploid gametes produced by octoploid or it is related to the functional genes? In order to address these questions, in this study, we used DH inducers parent P3–2 (2n = 4 × =38, AACC) [13], induction line Y3380 and Y3560, tetraploid, hexaploid and mixed-ploidy offspring of Y3380 and Y3560, and other polyploid (octoploid, hexaploid and triploids) were used as the pollen donors, and possible mechanism of DH induction was explored. The objective of this study was to establish a relationship between DH induction line with functional genes and ploidy, and how mechanism of DH induction provides a theoretical basis for our experiment.

## Results

### Somatic chromosome counting and meiotic analysis of parents

**Fig. 1:**
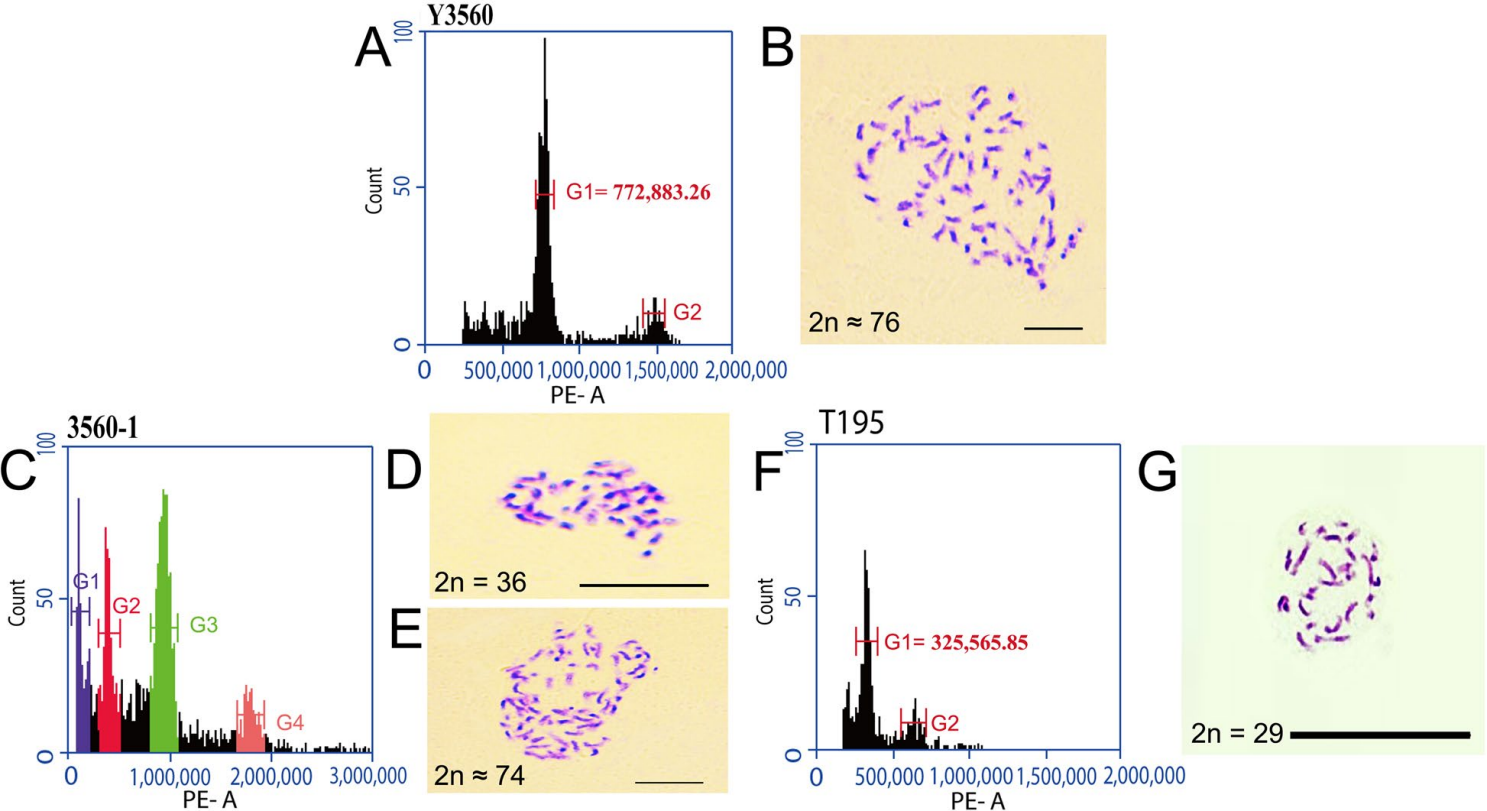
Material plant ploidy identification, and cytological observation. **a** Flow cytometry histogram of Y3560 plant. **b** Chromosome number of Y3560 plant. **c** Flow cytometry histogram of 3560–1 plant. **d** Chromosome number of 3560–1 plant. **e** Chromosome number of 3560–1 plant. **f** Flow cytometry histogram of T195 plant. **g** Chromosome number of T195 plant

The results of measuring the ploidy of the male and female parents by flow cytometry were in Additional file 1. Our results had shown that the number of somatic chromosomes of triploid, tetraploid, hexaploid and octoploid samples were 29, 38, 52–56 and 74–70 respectively. The fluorescence value of flow cytometry of triploid, tetraploid, hexaploid and octoploid samples were in between of 320,000 ~ 350,000D (Additional file 2), 390,000 ~ 450,000D (Additional file 3), 590,000 ~ 630,000D (Additional file 4) and 710,000 ~ 850,000D (Additional file 5), respectively. The results had shown two peaks were detected by flow cytometry (Fig. 1A), and the cells had 76 chromosomes in Y3560 (Fig. 1B). The sample 3560–1 from Y3560 isolated individual had shown that four peaks were detected by flow cytometry (Fig. 1C), and the cells had 36 and 74 chromosomes in same cell at the same time (Fig. 1D and E). The results indicated that it was a mixed-ploidy. The sample T195 had shown that two peaks were detected by flow cytometry (Fig. 1F), and the cells had 29 chromosomes (Fig. 1G), indicated T195 was triploid. The results of the number of chromosomes were consistent with the results of flow cytometry.

**Fig. 2:**
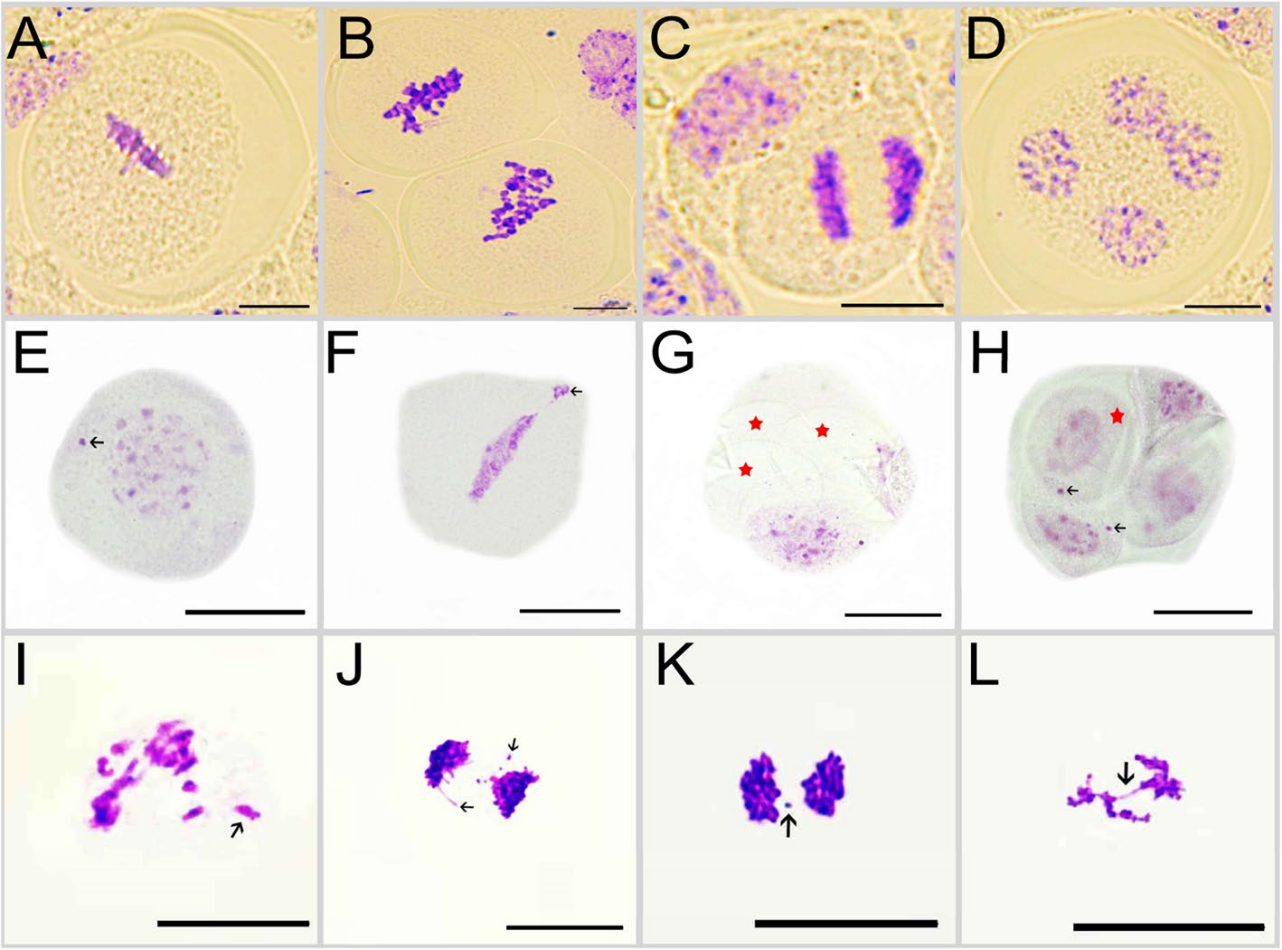
Observation of meiosis behavior. **a-d** Meiosis behavior of Y3560. **e-h** Meiosis behavior of 3560–1. **i-l** Meiosis behavior of T195. The arrow in the figure points to a lagging or extranuclear chromosome. The red five-pointed star is marked as defective empty spores. Scale bar: 10 μm

**Fig. 3:**
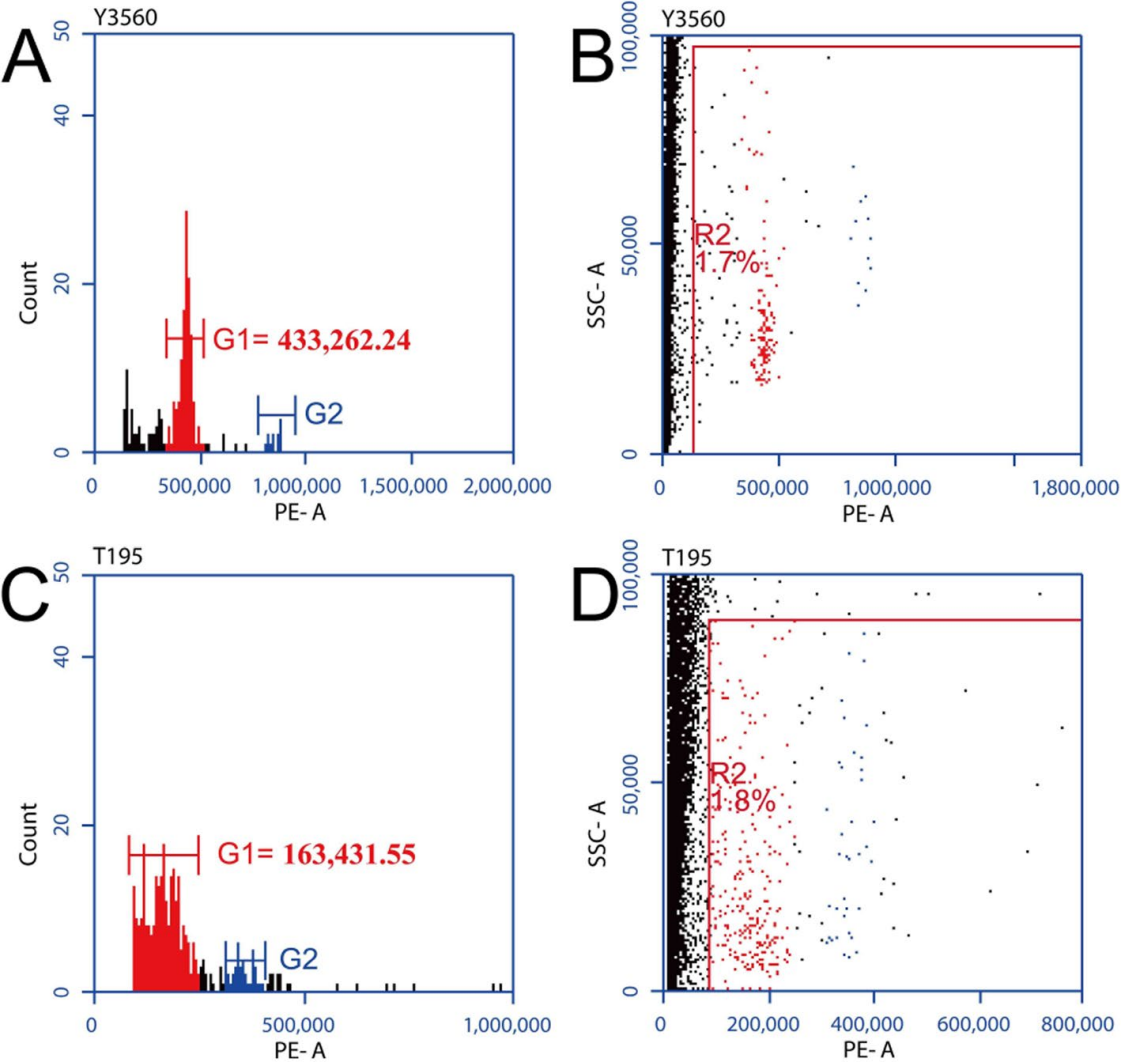
Pollen ploidy identification. **a-b** Flow cytometry histogram of Y3560 pollen. **c-d** Flow cytometry histogram of T195 pollen

Our microscopic observations indicate that the octoploid Y3560 has normal chromosome behavior in metaphase I and anaphase I (Fig. 2A-C), while chromosome division was not synchronized well at the tetrad stage (Fig. 2D). The mixed ploidy sample 3560–1 showed monovalent chromosomes in the cells of early stage I (Fig. 2E), large fragments of chromosomes appeared in metaphase I (Fig. 2F), while tetrad stage showed an obvious defective and empty spore (Fig. 2G). In addition, there were obvious monovalent chromosomes in the nucleus during the tetrad stage (Fig. 2H), which may easily produce aneuploidy gametes. The cells of the triploid sample T194 have lagging chromosome in early stage I and the late stage I (Additional file 6A, Additional file 6D), and the separation of large fragments of chromosomes (Additional file 6B, Additional file 6C). The cells of triploid T195 showed a phenomenon of laggard chromosome caused by chromosome mismatch at metaphase I (Fig. 2I), lagging chromosomes (Fig. 2 J and K) and chromosome bridges at later stage I (Fig. 2 L). Pollen flow cytometry results showed that Y3560 can form an obvious cell cycle (Fig. 3A and B), indicating that the pollens of Y3560 were good euploid gametes. But T195 can not form an obvious haploid peak and haploid cell population (Fig. 3C and D), and there were significant differences of single gametophyte cells produced by triploid with compared to Y3560 (Fig. 3B). The peak range of G1 (Fig. 3C) was more dispersed than that of euploid Y3560 (Fig. 3A). The results of meiosis were consistent with those of pollen flow cytometry, and indicated the aneuploid gametes were more likely to form after meiosis,because these chromosomes of triploid T195 can not be paired normally.

### Individual phenotypic identification and flow cytometric fluorescence measurement of F_1_ generation

**Fig. 4:**
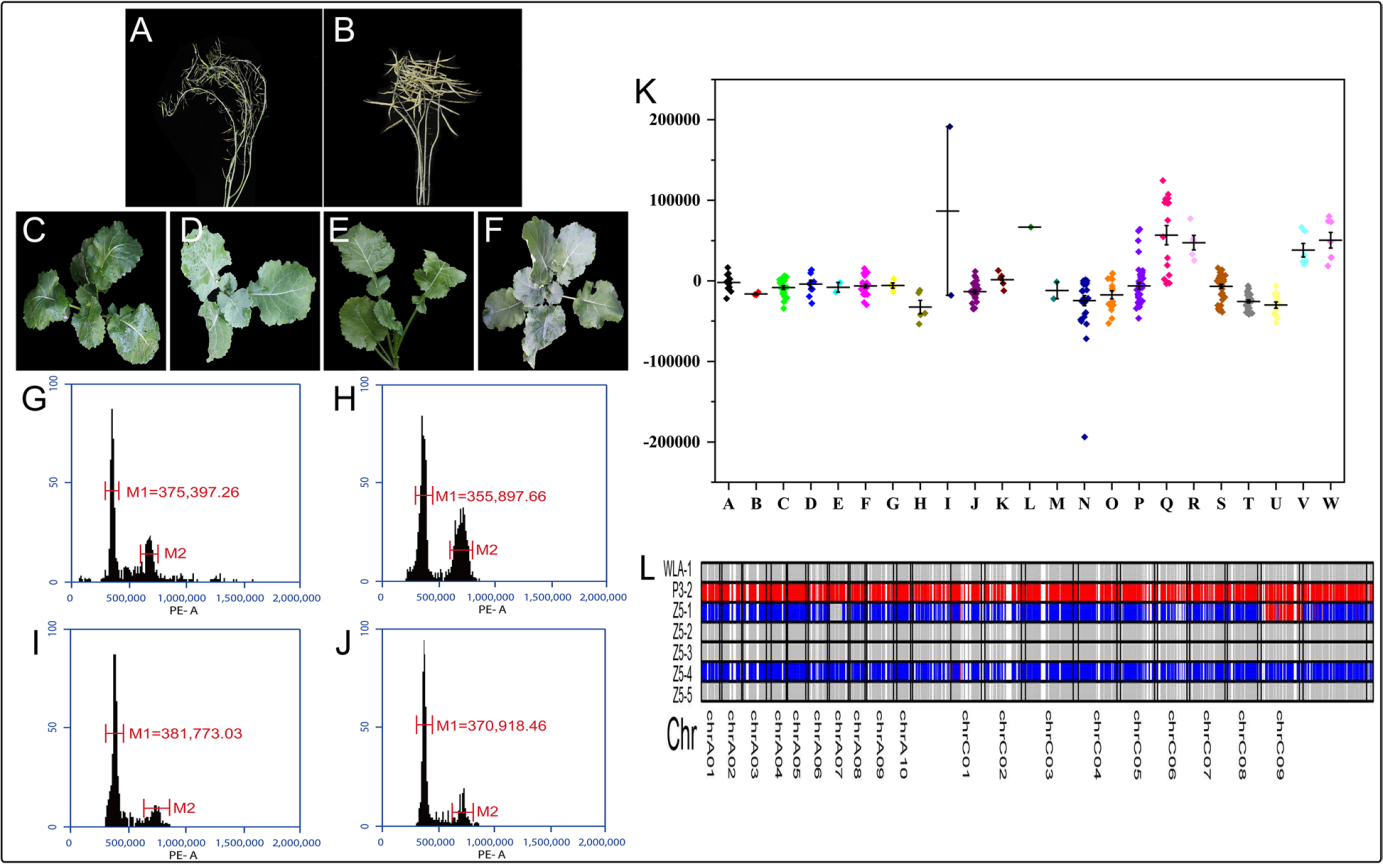
Identification of F_1_ generation individual. **a** W1A selfed seed setting. **b** F_1_ hybrid seed setting of WlA × Y3380. **c** Phenotype of female parent plant (WlA). **d** Phenotype of wax male parent (P3–2). **e** Phenotype of waxless double haploid F_1_ individual. **f** Phenotype of wax hybrid F_1_ individual. **g** Flow cytometry histogram of WLA. **h** Flow cytometry histogram of P3–2. **i** Flow cytometry histogram of waxless double haploid F_1_ individual. **j** Flow cytometer histogram of wax hybrid F_1_ individual. **k** Scatter plot of relative difference between F_1_ generation and maternal (W1A) flow cytometry fluorescence value. A-W successively are A: WlA × 3560–1, B: WlA × 3560–2, C: WlA × 3560–3, D: WlA × 3560–4, E: WlA × 3560–5, F: WlA × 3380–1, G: WlA × Y3380, H: WlA × Y3560, I: WlA × HZ23, J: WlA × HZ28, K: WlA × 3850, L: WlA × HZ1, M: WlA × HZ4, N: WlA × 3114, O: WlA × 3128, P: WlA × 3560–6, Q: WlA × 4417, R: WlA × DW39, S: WlA × P3–2, T: WlA × 3737, U: WlA × HZ24, V: WlA × T194D and W: WlA × T195. **l** F_1_ generation genotyping diagram of WlA-1 × P3–2

When the wax-less (WlA) *pol* cytoplasmic sterile line (CMS) was self-crossed, siliques were dyed and cannot produce normal seeds (Fig. 4A). However, the F_1_ generation siliques of the cross between W1A and the male parent (Y3380) were normal (Fig. 4B). So, sterile seed production by WlA by self-pollination was ruled out, when a different ploidy *B. napus* were crossed with WlA. So, WlA can be used as the female and there would be a need of reduced emasculation. In addition, the wax-less trait was being controlled by a recessive single gene, which enabled us to judge the phenotype of F_1_ generation plants easily. Therefore, WlA can be used as a tester line to directly observe whether the F_1_ generation is a hybrid (Fig. 4F) or a DH individual plant (Fig. 4E) from the morphology. The phenotypic results showed that the leaves surface of wax-less plants was smooth and darker green (Fig. 4C, E). Besides this, the leaves of the wax plants were fuzzy and grayish green (Fig. 4D, F). Through F_1_ generation plants, DH individual plants and hybrid plants can be preliminarily distinguished on the basis of leaf phenotype.

**Fig. 5:**
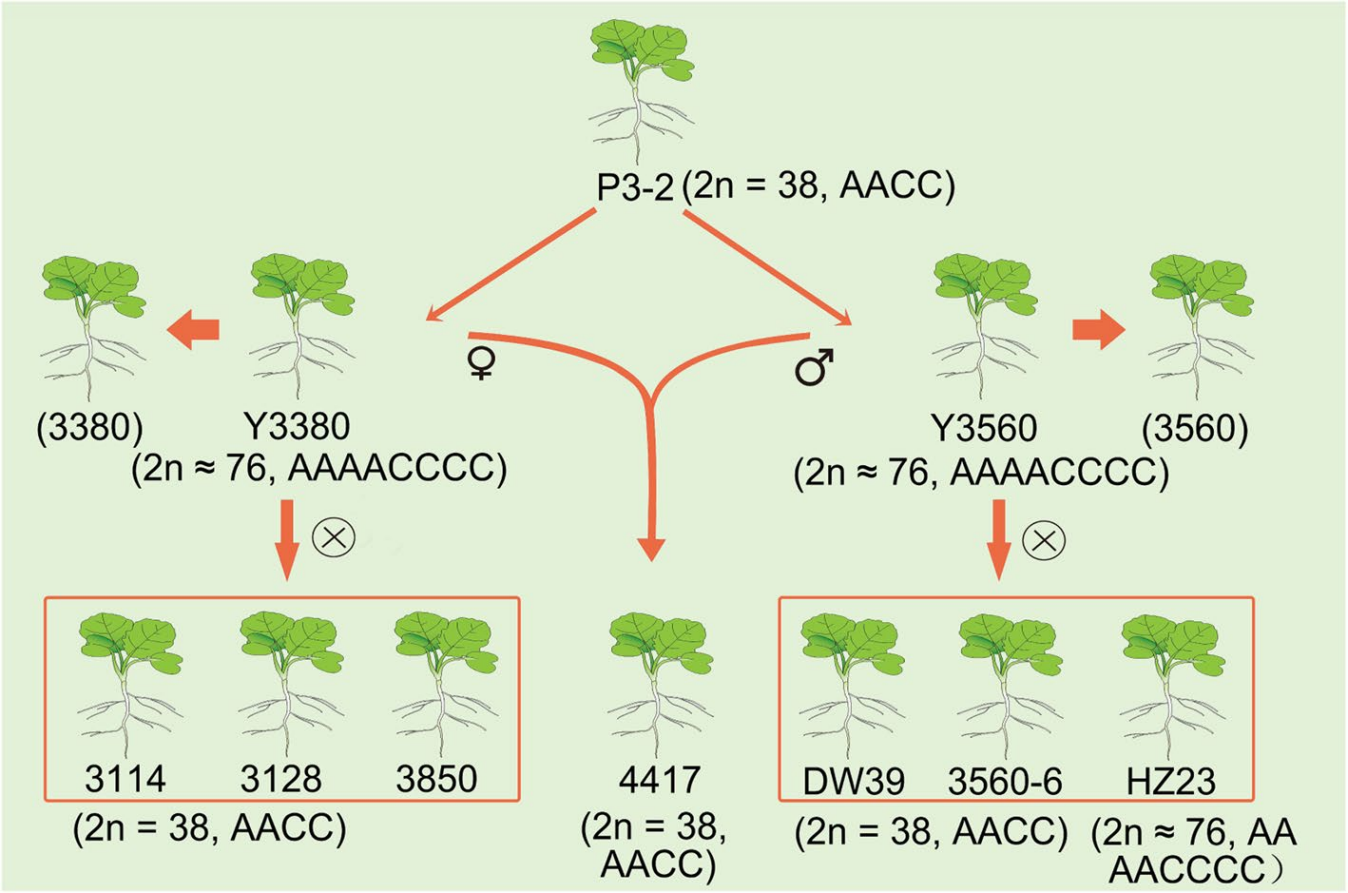
Sources of materials related to DH induction line

A total of 315 F_1_ individuals crossed in different hybrid combination were tested for the flow cytometry fluorescence values and waxy leaf phenotype (Additional file 7). Our study had shown that the flow cytometric fluorescence values of DH F_1_ individuals (Fig. 4I) induced by P3–2 was close to those of W1A (Fig. 4G). The difference in the fluorescence value of flow cytometry between individuals induced by P3–2 and hybrid individuals (Fig. 4 J) was small, because P3–2 (Fig. 4H) is a homozygous tetraploid *B.napus*, which is same as the ploidy of WlA. The F_1_ generation individual’s G1 flow cytometric fluorescence values of the 17 hybrid combinations include WlA × 3114, WlA × 3128, WlA × 3560–6, WlA × P3–2, WlA × 3737, WlA × HZ24, WlA × 3850, WlA × HZ4, WlA × 3560–1, WlA × 3560–2, WlA × 3560–3, WlA × 3560–4, WlA × 3560–5, WlA × 3380–1, WlA × Y3380, WlA × Y3560 and WlA × HZ28 are lower than those of W1A (Fig. 4 K, Fig. 5). The F_1_ generation individual G1 flow cytometric fluorescence values of the six hybrid combinations include W1A × 4417, W1A × DW39, W1A × HZ1, W1A × HZ23, W1A × T194 and W1A × T195 are higher than those of W1A (Fig. 4 K). Since the female parent WlA is a homozygous tetraploid *B.napus*, the flow cytometric fluorescence value of the WlA individual in the G1 phase is relatively stable, so it is speculated that the fluorescence value of the F_1_ generation G1 phase of different combinations may be related with the male’s DNA content and the paternal chromosome infiltration. F_1_ generation individual G1 phase flow cytometric fluorescence values of W1A × HZ23 are quite different, which is presumably the uneven meiosis of hexaploid HZ23 due to production of different ploidy gametes. In addition, a haploid individual is found in the offspring of W1A × 3114 (No.28 single plant, 1/33, 3.03%, Additional file 7), indicating that 3114 may have the ability to induce the offspring to produce haploid plant. At the same time, we combine the flow cytometry to determine the F_1_ generation individual G1 phase flow cytometry results to verify the ploidy of some F_1_ generations (Additional file 8). The number of chromosomes observed by somatic cell microscope was consistent with the ratio of G1 phase fluorescence signal detected by flow cytometry (Additional file 3, Additional file 4, Additional file 9), which indicates that flow cytometry can accurately determine the ploidy of hybrid or induced offspring individual.

### Identification of homozygote or heterozygote in F_1_ samples

In order to ensure that the SNP chip analysis is true representative, the 28,491 effective sites were detected and plotted on the SNP density distribution map on the 19 chromosomes (Additional file 10). SNP detection sites were more evenly distributed on A and C chromosome, and can be used for subsequent analysis. In this study, the F_1_ generation individuals were preliminarily judged on the basis of phenotypes e.g., with or without of wax, coupled with results of flow cytometry. In order to verify its reliability of the phenotype and the ploidy analysis, representative individuals were selected to identify whether F_1_ generation is homozygote and heterozygote based on SNP chip analysis (Table 1). The 28,491 effective sites were detected, classified and counted, which are AA, BB, AB, and NA. The average SNP homozygous sites rate of WlA (female) homozygous lines with continuous backcrossing for more than 10 generations was 93.17%. Therefore, the SNP homozygous site rate of 93.17% was used as standard to judge whether the offspring were homozygous or not. Results of SNP homozygosity rate shows that 3737 (tetraploid) had no inducibility and the F_1_ generations obtained are all hybrid individuals. The SNP homozygous site rates of the F_1_ hybrid individuals Z16–1 and Z16–2 were tested, and the results showed that the SNP homozygous site rates of these two individual plants were 64.57 and 64.16%, respectively (Table 1). Therefore, the homozygous site rate of 64.37% is used as the standard to judge whether the offspring are heterozygous or not. The analysis results showed that among the 64 representative individuals, 17 individuals were found with wax and one individual (Z19–1) without wax were heterozygous, and the homozygous site rate was 59.71 ~ 67.01% (Table 1); The remaining 46 individuals without wax were homozygous, and the rate of homozygous site was 92.72 ~ 98.70% (Table 1). SNP chip identification shows that, except for Z19–1, all individuals without wax were homozygous. Z19–1 is a wax-less individual, but the SNP chip identified it as a heterozygote. It could be due to the recessive gene controlling waxiness, as probability of waxiness trait occurrence was only one plant (1/47, 2.13%). Therefore, it is proved that the results of phenotypic identification and flow cytometry can identify the homozygotes or heterozygotes of F_1_ generation individuals.

**Table 1:** Summary of individual SNP chip identification in F_1_ generation

Sample number	With or without wax	Value of Fluorescence flow cytometry	Rate of homozygous site (%)	Homozygous and different site number of parents	Number of sites same as the female parent	Male parent infiltration rate (%)	Hybridization site rate (%)
Z1–1	No	380,167.50	93.43	6377	6361	0.00	0.00
Z1–2	No	402,510.90	93.29	6377	6364	0.00	0.00
Z1–3	No	357,577.39	93.31	6377	6370	0.00	0.00
Z1–4	No	393,843.57	93.18	6377	6359	0.00	0.03
Z1–5	No	379,213.62	93.25	6377	6368	0.00	0.00
Z1–6	No	370,379.61	93.35	6377	6360	0.00	0.02
Z1–7	No	396,666.50	93.52	6377	6360	0.00	0.00
Z2–1	No	379,647.85	93.71	6569	6551	0.20	0.00
Z2–2	No	403,773.06	93.65	6569	6550	0.20	0.00
Z2–3	No	365,186.36	92.93	6569	6499	0.00	0.06
Z3–1	No	369,954.25	93.66	6660	6649	0.00	0.00
Z3–2	No	373,199.38	93.64	6660	6649	0.00	0.00
Z3–3	No	370,213.29	93.61	6660	6649	0.00	0.00
**Z4–1**	**Yes**	**380,411.61**	**59.71**	**8147**	**356**	**0.64**	**92.91**
Z4–2	No	358,807.06	93.23	8147	8120	0.00	0.01
Z4–3	No	366,565.08	93.35	8147	8135	0.00	0.00
**Z5–1**	**Yes**	**356,672.96**	**62.73**	**9233**	**324**	**3.58**	**90.40**
Z5–2	No	370,918.46	93.37	9233	9224	0.00	0.00
Z5–3	No	397,723.89	93.60	9233	9218	0.00	0.01
**Z5–4**	**Yes**	**403,230.98**	**60.77**	**9233**	**64**	**0.41**	**96.64**
Z5–5	No	384,689.82	93.63	9233	9220	0.00	0.00
Z6–1	No	375,084.27	93.61	9130	9101	0.00	0.16
Z6–2	No	390,668.40	93.23	9130	9087	0.01	0.07
Z6–3	No	384,049.43	93.49	9130	9095	0.24	0.00
Z7–1	No	454,023.50	92.72	10,469	10,409	0.00	0.43
Z8–1	No	385,795.47	93.66	6730	6719	0.09	0.00
Z8–2	No	365,029.51	93.60	6730	6721	0.09	0.00
**Z9–1**	**Yes**	**578,710.61**	**64.61**	**7236**	**3008**	**0.11**	**46.41**
**Z9–2**	**Yes**	**369,157.11**	**66.73**	**7236**	**3070**	**4.99**	**50.72**
Z10–1	No	359,277.98	98.70	9338	9308	0.01	0.09
**Z10–2**	**Yes**	**365,940.57**	**60.45**	**9338**	**56**	**0.51**	**97.34**
**Z10–3**	**Yes**	**319,310.70**	**60.76**	**9338**	**52**	**0.82**	**96.84**
Z11–1	No	375,536.34	93.54	6972	6917	0.65	0.01
Z11–2	No	333,786.55	93.52	6972	6920	0.65	0.01
Z11–3	No	345,056.30	93.52	6972	6919	0.65	0.01
Z12–1	No	385,472.76	93.53	8364	8315	0.30	0.19
**Z12–2**	**Yes**	**315,531.95**	**63.54**	**8364**	**75**	**0.84**	**96.32**
Z12–3	No	368,941.21	93.62	8364	8326	0.29	0.00
Z13–1	No	379,745.28	93.07	7731	7650	0.52	0.26
Z13–2	No	373,948.74	93.29	7731	7687	0.14	0.27
Z13–3	No	389,945.59	92.93	7731	7664	0.04	0.72
**Z14–1**	**Yes**	**362,004.55**	**62.72**	**8801**	**50**	**1.59**	**96.17**
**Z14–2**	**Yes**	**334,489.64**	**62.15**	**8801**	**54**	**1.00**	**96.74**
**Z14–3**	**Yes**	**396,302.44**	**62.23**	**8801**	**58**	**0.97**	**95.40**
**Z15–1**	**Yes**	**494,644.08**	**67.01**	**8163**	**50**	**10.62**	**86.49**
**Z15–2**	**Yes**	**442,013.75**	**66.64**	**8163**	**3846**	**4.47**	**46.00**
**Z15–3**	**Yes**	**394,262.46**	**64.85**	**8163**	**3983**	**0.28**	**49.04**
**Z16–1**	**Yes**	**355,685.45**	**64.57**	**7606**	**102**	**0.75**	**95.24**
**Z16–2**	**Yes**	**299,415.21**	**64.16**	**7606**	**102**	**0.76**	**95.15**
**Z17–1**	**Yes**	**464,444.93**	**62.49**	**8373**	**50**	**0.74**	**94.88**
Z17–2	No	439,435.82	93.52	8373	8330	0.26	0.01
Z17–3	No	412,492.42	93.51	8373	8333	0.26	0.01
Z18–1	No	365,742.23	93.40	6156	6105	0.47	0.15
Z18–2	No	374,924.44	93.16	6156	6105	0.47	0.18
Z18–3	No	352,318.10	93.15	6156	6090	0.47	0.41
Z18–4	No	398,995.37	93.19	6156	6086	0.83	0.08
**Z19–1**	**No**	**650,382.52**	**62.40**	**7881**	**3900**	**0.38**	**48.67**
Z19–2	No	643,543.85	93.20	7881	7835	0.03	0.11
Z19–3	No	600,221.32	93.20	7881	7817	0.05	0.33
Z19–4	No	590,485.00	93.20	7881	7810	0.37	0.14
Z20–1	No	660,128.07	93.40	7683	7611	0.49	0.00
Z20–2	No	661,356.33	93.20	7683	7606	0.49	0.00
Z20–3	No	596,629.91	93.30	7683	7609	0.46	0.03
Z20–4	No	596,905.70	93.10	7683	7607	0.46	0.03

In the table, bold represent the individual is heterozygous, and others represent the individual is homozygous. Z1 ~ Z20: F_1_ generation individuals of WlA × 3380–1,WlA × 3560–1,WlA × 3560–2,WlA × 3560–6,WlA × P3–2,WlA × 3850,WlA × HZ1,WlA × HZ4,WlA × HZ23, WlA × HZ24, WlA × Y3560, WlA × 3114, WlA × Y3380, WlA × 3128, WlA × 4417,WlA × 3737, WlA × DW39, WlA × HZ28, WlA × T194, WlA × T195, respectively

The cluster analysis of 64 F_1_ generation individuals (Additional file 16) were consistent with the results of SNP chip identification of homozygotes and heterozygotes. Compared with the male parent, the genetic distance between the F_1_ generation and the female parent (WlA) was relatively closer. In general, the F_1_ generation population and WlA were placed in same cluster class (Additional file 11). Compared with F_1_ generation homozygous individuals, the genetic distance between F_1_ generation heterozygote and W1A is farther, and the 17 heterozygotes and Z19–1 individual identified by the SNP homozygous site rate were clustered in two classes e.g., C1 and C2. The genetic distance between F_1_ homozygous and WlA is relatively closer. Forty-six homozygous and WlA identified by the SNP homozygous site rate were clustered in class D (Additional file 11). The results of cluster analysis assist the reliability of SNP homozygous loci rate.

### SNP genotyping analysis of F_1_ generation

Due to the high homozygous rate of WlA, the genetic distance among WlA-1, WlA-2, and WlA-3 individual plants was very close. Therefore, in the genotyping analysis, the F_1_ generation individual Z1 corresponds to the female parent W1A-2, the F_1_ generation individual Z4 resembles to the female parent W1A-3, and the other F_1_ generations was corresponded to the female parent W1A-1. There were obvious differences in male infiltration rate of F_1_ heterozygous individuals. The hybridization site rate of 18 heterozygous samples was 46.00 ~ 96.84% (Table 1). The Z9–1, Z9–2, Z15–1, Z15–2 and Z19–1 have more than 3000 identical sites with the female parent W1A (Table 1), which were significantly higher than other hybrid individuals. The hybridization rate of 46 homozygotes was 0 ~ 0.72%, and the male parent’s infiltration rate was 0 ~ 0.83% (Table 1), which indicates that the induction effect was higher and male parent’s infiltration rate and hybridization site rate was smaller.

### Relationship between induction efficiency and male paternal ploidy

Combining phenotype identification, G1 flow cytometry ploidy determination and SNP chip analysis can be used to verify F_1_ DH individuals. Because triploid, hexaploid and octoploid meiosis would undergo unbalanced division, which leaded to the appearance of different ploidy or aneuploid gametes. If the phenotype of crossing F_1_ individuals of W1A and triploid, tetraploid, hexaploid, octoploid rapeseed is identified as wax-less, ploidy determined by flow cytometry is tetraploid, and the SNP chip identified it as homozygous, it would be a DH individual. The rest are considered as F_1_ hybrids.

This study had shown that there was a significant difference in the induction rate among the sample. The induction rate of mixed ploidy, octoploid, hexaploidy, tetraploid and triploid samples were 50.00 ~ 100%, 0 ~ 100, 100%, 0 ~ 92.02%, and 83.33 ~ 100%, respectively (Table 2). Such variations suggest that the induction is not entirely caused by the difference of parental ploidy, but may be related to the functional genes of the male parent.

**Table 2:** Different ploidy material induction efficiency

Cross combination	Paternal ploidy	Effective seed setting rate (number of seeds/(number of effective siliques*25))%	Number of F_1_ generation survived plants/number of seeds (%)	Number of waxless plants/total number of plants	Number of tetraploid plants/total number of plants	Induction rate (%)	Selfing male parent survival rate (%)
WlA × 3560–1	Mixed ploidy	(69/(19*25))14.53	12/69(17.39)	12/12	12/12	100.00	17/100(17.00)
WlA × 3560–2	Mixed ploidy	(75/(20*25)) 15.00	3/75(4.00)	3/3	3/3	100.00	7/78(8.97)
WlA × 3560–3	Mixed ploidy	(119/(28*25)) 17.00	23/100(23.00)	23/23	23/23	100.00	NA
WlA × 3560–4	Mixed ploidy	(108/(27*25)) 16.00	9/100(9.00)	7/9	9/9	77.78	NA
WlA × 3560–5	Mixed ploidy	(8/(5*25)) 6.40	8/25(25.00)	1/2	2/2	50.00	24/76(31.58)
WlA × 3380–1	Mixed ploidy	(100/(36*25)) 11.11	22/100(22.00)	22/22	22/22	100.00	56/80(70.00)
WlA × Y3560	Octoploid	(35/(10*25)) 14.00	5/35(14.29)	5/5	5/5	100.00	NA
WlA × Y3380	Octoploid	(332/(35*25)) 37.94	4/100(4.00)	4/4	4/4	100.00	NA
WlA × HZ23	Octoploid	(5/(4*25)) 5.00	2/5(40.00)	0/2	1/2	0.00	12/14(85.71)
WlA × HZ28	Octoploid	(198/(40*25)) 19.80	25/100(25.00)	124/125	25/25	98.67	0/0(0.00)
WlA × 3850	Hexaploid	(62/(65*25)) 3.82	5/62(8.06)	5/5	5/5	100.00	12/100(12.00)
WlA × HZ1	Hexaploid	(21/(10*25)) 8.40	1/21(4.76)	1/1	1/1	100.00	26/100(26.00)
WlA × HZ4	Hexaploid	(24/(14*25)) 6.86	2/24(8.33)	2/2	2/2	100.00	10/100(19.00)
WlA × 3114	Tetraploid	(915/(45*25)) 81.33	33/100(33.00)	16/133	33/33	8.02	NA
WlA × 3128	Tetraploid	(1303/(66*25)) 78.97	16/100(16)	0/116	16/16	0.00	NA
WlA × 3560–6	Tetraploid	(684/(69*25)) 39.65	64/100(64)	129/139	32/39	92.02	7/11(63.64)
WlA × 4417	Tetraploid	(720/(37*25)) 77.84	16/100(16.00)	0/116	16/16	0.00	NA
WlA × DW39	Tetraploid	(971/(59*25)) 65.83	11/100(11.00)	8/105	2/5	17.33	7/100(7.00)
WlA × P3–2	Tetraploid	(221/(59*25)) 14.98	37/100(37.00)	11/138	38/38	7.96	7/32(21.88)
WlA × 3737	Tetraploid	(956/(47*25)) 81.36	26/100(26.00)	0/126	26/26	0.00	24/100(24.00)
WlA × HZ24	Tetraploid	(216/(64*25)) 13.50	14/176(7.95)	6/14	14/14	42.86	20/100(20.00)
WlA × T194	Triploid	NA	NA	6/6	6/6	83.33	0/0(0.00)
WlA × T195	Triploid	NA	NA	7/7	7/7	100.00	0/0(0.00)

In the table, T194 has a heterozygous phenotype with no wax powder, so the induction rate is 83.33%

*NA* not available

Compared with other high-ploid rapeseeds, DH induction lines e.g., Y3380 and Y3560 can not only selfing to produce seeds [17], but also genetically regulate the genes of inducibility. Analysis of variance showed that the induction rates of 3560–6, HZ28 and DW39 were significantly higher (*P* < 0.05) than 3737; while the induction rates of 3114, 3128, 4417 and P3–2 were not significantly different from 3737 (Additional file 12). It shows that the induction effect of Y3560 separated offspring (3560–6 and DW39) is better than that of Y3380 separated offspring (3114 and 3128). Moreover, induction efficiency data had shown that the male parent survival rate of DH induction line for selfing and DH induction line was 7.00 ~ 85.71% and 0 ~ 24%, respectively (Table 2). Although some male parents can induce DH induction, but their seed setting rate were lower or sometimes they were even unable for self-fertilization at all. Application value of those DH inducers is low. For example, the induction rates of HZ28, T194 and T195 were 98.67, 83.33 and 100% respectively, indicating that they have the ability to induce, but the survival rate of selfing was 0%. In addition, some male parents (H1 and H4) have induction ability, but the total number of plants in F_1_ generation and the effective seed setting rate were too low. Correlation analysis showed that the effective seed setting rate was positively correlated with the survival rate of F_1_ generation (r = 0.143, *P* = 0.538, *N* = 21). There were some cases, where the seeds were healthy but their offspring was not vigorous.

## Discussion

### Effect of genotypes on inducibility of DH inducer in *Brassica napus* L

Studies on the effects of functional genes of DH induction are progressing slowly than other molecular studies. At present, three genes have been identified that can induce DH induction, *CENTROMERIC PROTEIN* (*CENH3)*, maize *POLLEN-SPECIFIC PHOSPHOLIPASE A GENES* (*ZmPLA1*) and *ZmDMP*. In unstable hybrid embryos, the CENH3 centromeric protein could not be successfully integrated into the centromere of one of the parents, resulting in the disappearance of the a parent chromosome [7]. The loss of function caused by the 4 bp insertion of the *ZmPLA1* orthologous to MATRILINEAL (MTL*,* also known as *ZmPLA1* and NLD) is the key gene for induction in maize haploids [12]. The homologous genes of *ZmPLA1* in wheat and rice have been edited to obtain mutants that can induce haploid production from the maternal parent, indicating that haploid production may have the same recognition mechanism in different species [18, 19]. CAUHOI and CAU5 match the *ZmDMP* gene via map-based cloning. The single-base mutation can increase the haploid induction rate by 2 ~ 3 times, and the complete knockout could increase the induction rate by 5 ~ 6 times [11]. In our research, we use with different ploidy rapeseed as the male parent to pollinate the wax-less female parent (WlA) of the tester species. The results showed that not all rapeseed materials can induce DH individual production. As the core parent of DH induction lines, P3–2 has inducibility (Table 2, induction rate 7.96%). DH inducer lines Y3380 and Y3560 showed induction, but DH inducer Y3380 inbred progeny (3128) and DH inducer Y3560 inbred progeny (HZ23) have no induction, confirms the hypothesis that functional genes regulate the inducibility of DH parents and provide theoretical support for P3–2 to construct a population and locate the functional genes that regulate the inducibility. At the same time, HZ28, HZ1, HZ4, HZ24, and T195 had the inducibility, which are not DH induction lines, indicating that the phenomenon of inducing DH individuals is not unique to DH induction line. Those rapeseed materials that were not DH induction line have other genes or other regulatory mechanisms that have the ability to regulate induction. In addition, although HZ28, T194 and T195 had inducibility, the survival rate of selfing is low (0%), and stable inheritance cannot be achieved. Studies reported that DH induction lines have been self-fertilized for about 10 generations, with a high survival rate of selfing and most of their offspring are genetically stable [17] .

After a species has undergone a genome-wide replication event, it often produced some new traits and functions [20] . The synthetic polyploid genome structure is unstable and prone to rapid changes [21]. For example, the selfing offspring of allo-hexaploid of synthetic *Brassica* often suffered from chromosome loss, producing in a series of offspring with different numbers of chromosomes, but eventually it stabilizes in the tetraploid [22]. Stable inheritance of repeat-associated siRNAs maintains chromatin and genome stability, whereas expression variation of miRNAs leads to changes in gene expression, growth vigor, and adaptation [23]. The artificially synthesized *Brassica napus* undergoes rapid changes in the genome structure, including the loss of parental fragments [24]. Genomic stress in synthetic rapeseeds could disrupt the DNA methylation balance of the species and disturb the regulation of DNA damage repair, nucleotide metabolism, and the cell cycle, which in turn would increase the instability and fragility of polyploid rapeseed genomes [25]. The DH induction lines (Y3380, Y3560) in this experiment are allo-octoploids, and the selfed progeny of Y3380 isolated 3114 (tetraploid), 3128 (tetraploid) and 3850 (hexaploid). Three thousand one hundred twenty-eight has no inducibility, while 3114 and 3850 have inducibility and the induction ability of 3560–6 (tetraploid) from Y3560 isolated offspring reached to 92.02%. HZ23 (octoploid) from Y3560 selfing offspring and the 4417 (tetraploid) from the cross between Y3380 and Y3560 also have neither induction ability. Presumably because the induction line is a synthetic octoploid rapeseed, during the selfing process, the parental chromosome fragments are lost due to rapid diploidization. At the same time, transposon silencing, chromosome recombination, and partial homologous chromosome pairing occurred [26–28], resulting in the loss or remodeling of functional genes that regulate the inducibility, which eventually led to differences in inducing function among 3114, 3128, 3850, 3560–6 and 4417.

Polyploidization events can cause species to double at the genome level, and increase in gene copy number usually lead to changes in the gene level [29]. The possible factors that cause changes in polyploid gene expression including gene dosage effects, changes in gene expression regulatory networks, rapid genetic and epigenetic changes [30]. In theory, the increase in the copy number of all genes has the same effect on all genes, and should lead to a consistent increase in gene expression, which is the additive effect of genes. However, not all genes in polyploidy show simple additive effects. A certain proportion of non-additively expressed genes are often detected in synthetic polyploids of wheat, arabidopsis and cotton [31–33]. In this study, induction rate of DH line Y3380 (octoploid), Y3380 inbred progeny 3850 (hexaploid), 3114 (tetraploid) and P3–2 (tetraploid) are 100, 100, 8.02 and 7.96%, respectively. It can be seen that the induction rate of hexaploid and octoploid samples is significantly higher than that of tetraploid samples P3–2 and 3114. Therefore, considering the induction rate, we believe that due to the doubling of the samples at genome level, the copy number of functional genes that regulate the inducibility is increased, and there is a certain degree of gene additive effect, which led to a significant increase in the induction rate of double haploid induction lines.

### Effects of aneuploidy on DH induction lines

Use of DH can speed up the breeding process and can save a lot of budget. At present, haploid technology is being used in corn (*Zea mays* L*.*), wheat (*Triticum aestivum* L*.*), barley (*Hordeum vulgare* L*.*), rapeseed (*Brassica napus* L*.*), tobacco (*Nicotiana Tabacum* L*.*), etc. The disappearance of parental chromosomes in the early embryonic development is the reason for interspecific/intraspecific hybridization to produce haploid plants [34]. The specific mechanism of action is not clear, but there are many hypothesis which include non-synchronization of cell cycle events [35], parental genome imbalance [36], sister chromatid division fault, etc. [37]. The common feature of related hypothesis is that plants recognize themselves and respond to foreign DNA in a variety of ways, and then try to get rid of them. Studies have also shown that through microspore single cell sequencing, it was found that the induction of maize haploid may be related to pollen aneuploidy [17]. After whole-genome duplication (WGD), species chromosomal instability is widespread, and aneuploidy is common in early generations of new polyploid [38, 39]. Due to the gene-dosage imbalance of aneuploid individuals, they often exhibit serious phenotypic defects (aneuploid syndrome), such as developmental delay, short individuals, and difficulty in reproducing offspring. Therefore, in most cases, aneuploidy is fatal to animals, humans, but plants often show strong tolerance to aneuploidy, especially in allopolyploid plants. Aneuploidy is very common in natural hexaploid wheat, probably because of its hetero-hexaploid characteristics that make it more tolerant of the loss or gain of chromosomes or chromosome arms [40]. Studies have found that incomplete homologous chromosome pairing leads to aneuploidy in hexaploid wheat [41]. Researchers have also found that the appearance of mixed progeny proves the reason for the doubling of early haploid maize [42]. In addition, mixed-ploidy plants may play a bridge role in the formation of plants with different ploidy levels [43]. Although mixed-ploid plants have been reported in *Brassica napus*. The effects of mixed-ploid plants on the genomic diversity and ploidy variation of *Brassica napus* has not been reported yet. In this experiment, based on the research that the induction of maize haploids may produce chromosomal fragments [17]. It was reported in previous studies that the DH induction line of mixed-ploid rapeseed is detected by flow cytometry. By observing the number of chromosomes in mitosis, it was detected that the number of chromosomes in different cells in the same plant was significantly different, indicating that the results of flow cytometry detection of mixed-ploid plants were reliable. Subsequently, we observed the meiotic behavior of mixed-ploid DH induction line (3560–1), and found that it is prone to form extranuclear chromosomes during the tetrad period (Fig. 2H), which led to an increased probability of producing aneuploid gametes. SNP genotyping analysis showed that the F_1_ generation Z1, Z2, and Z3 hybridization site rate and male infiltration rate of hybrid rapeseed DH induction lines (3380–1, 3560–1, 3560–2) are 0 ~ 0.06% and 0 ~ 0.2%; The F_1_ generation Z11 and Z13 hybridization sites and male infiltration rates of DH induction lines (Y3560, Y3380) are 0.01 ~ 0.72% and 0.04 ~ 0.65%. Compared with mixed-ploid DH induction lines (3380–1, 3560–1, 3560–2), the DH induction lines (Y3560 and Y3380) reduced the male infiltration rate of F_1_ and hybridization site rate, so that the offspring is not easy to infiltrate the paternal gene (Table 1). The occurrence of aneuploidy would reduce the male infiltration rate and the hybridization sites of induced F_1_ generation. Combined with the observation of meiosis behavior, it was speculated that the mixed-ploid DH induction line is prone to form extranuclear chromosomes due to the meiotic tetrad period (Fig. 2H), which increases the probability of aneuploid gametes. However, the formation of non-euploid gametes is unstable, and chromosomes are more likely to be lost during the later stage of the fusion of male and female gametes, which leads to a decrease in the male infiltration rate of F_1_. Therefore, when selecting DH inducers to quickly homozygous superior varieties in the future, we can choose mixed-ploid DH inducers for induction. While shortening the breeding process, excellent varieties with low male infiltration sites and low hybridization sites are obtained. The induction rate of mixed-ploid double haploid induction lines was 50 ~ 100%, and the induction rate of double haploid induction lines Y3380 and Y3560 was 100%. Regardless of whether the DH inducer produces aneuploid gametes or not, there may be functional genes that regulate the inducibility. Therefore, it has not been observed that the mixed-ploid and euploid DH inducers have significant changes in induction rate. At the same time, the synthetic triploid T195, which has nothing to do with the rapeseed DH induction line, is proved by meiosis observation that it can produce aneuploid gametes. The identification of the F_1_ generation of T195 showed that T195 can induce the female parent to produce DH individuals, which proved that the production of aneuploid gametes from pollen may be an important reason for the induction of rapeseed. P3–2 chromosome behavior had chromosomal backwardness (Additional file 13). It was examined that there may be genes controlling pollen generation aneuploidy in the parent P3–2 of the double haploid induction line. Whether it is the same as the *ZmPLA1* and *ZmDMP* remains to be further analyzed.

### Preliminary analysis of the induction process of DH induction lines

The common method of doubling haploids is treatment with colchicine, in addition to spontaneous chromosome doubling and direct induction of DH lines. The probability of maize induction lines directly inducing EH (Early Haploid Doubling) is very low (about 2%), and it was not enough to achieve the reported average spontaneous haploids doubling rate [44–46]. In this experiment, the induction rate of rapeseed is 7.96 ~ 100%, which is significantly higher than the previously reported maize induction line [42]. Various spontaneous doubling mechanisms may lead to the production of EHs. The elimination of chromosomes may make the chromosomes of the receptor cell “instability”, leading to “internal division” of the chromosomes themselves, and leading to failure of the mitotic spindle assembly. There are two possible ways for EH plants to appear, according to first female parent material produces 2n female gametes through abnormal meiosis during the induction process and develops into diploid through parthenogenesis [47, 48]. There are many abnormal factors of meiosis that lead to the formation of 2n gametes, such as meiotic nucleus restoration, secondary division spindle healing and abnormal cytokinesis. The previous studies have found that ectopic expression of *BBM1* in egg cells can induce parthenogenesis in rice, and use “mitosis instead of meiosis” to establish a rice apomictic reproduction system, and extend this method to most cereal crops [49]. Ionizing-irradiated pollen can induce parthenogenesis in Walnut and Styrian Pumpkin to produce haploids [50–52]. Second possibility, EH can be produced from normal n-gametes or haploid zygotes produced during the hybridization process of maternal materials and inducible lines. During subsequent seed development, the embryo’s genome doubles through abnormal mitosis [47, 48]. Studies have also found that 4C content of DNA appears during the fusion of male and female gametes in plants [53]. In this study, through SNP genotyping analysis of F_1_ generation individuals induced by rapeseed, homozygous and heterozygous F_1_ generations are identified (Fig. 4 L, Table 1). Genotyping graph analysis finds that the induced DH individual infiltration and hybridization sites have a certain degree of specific insertion on the ChrC03 chromosome (Additional file 14), indicating that the insertion of the paternal chromosome fragment during the induction process may be specific instead of random insertion. The hybridization rate of homozygotes individuals and the paternal infiltration rate was 0 ~ 0.72%, and 0 ~ 0.83%, respectively. It proves that the induction of offspring is not a simple parthenogenesis. It was speculated that normal n gametes or haploid fertilized eggs are produced during the hybridization induction process, and the male gametes combine with it and develop into diploids during the subsequent seed development process. At the same time, the paternal genome is partially or completely. Recently, Li Chao et al. Used the genome editing system mediated by the double haploid inducer Y3380 to modify the multigene homologous sequences of *Brassica napus* and *Brassica napus* directly, which also proved that the parernal chromosome was selectively eliminated after the formation of zygote. And the parernal chromosome released the editing specific sequence to edit the maternal gene, there was no homozygous editing site in the editing offspring, It suggests that editing may occur after chromosome doubling of female parent [15]. The natural doubling of chromosomes may be the characteristic of *Brassica* [54, 55], not the result of inducing genes, because triploids (T194 and T195) which are easy to produce aneuploid gametes can also induce DH progenies.

## Conclusions

At present, there are few studies on the induction mechanism of DH induction lines. In this study, it was found that functional genes regulate the inducibility of DH induction lines, and provided an experimental basis for the subsequent location of genes that regulate the inducibility of rapeseed. At the same time, we evaluated the effect of genome doubling on the induction rate of DH induction lines and their offspring. Our findings showed that doubling the genome can increases the induction rate of DH induction lines to a certain extent. However, the octoploid *Brassica napus* has high ploidy and abnormal meiotic chromosomal behavior. The tetraploid obtained by self-segregation has obvious changes in the induction function. How to achieve stable inheritance of octoploid *Brassica napus* is essential prerequisite of DH breeding program. In addition, the production of aneuploid gametes by pollen may be an important reason for inducing ability of rapeseed production. We also observed that normal n gametes or haploid fertilized eggs are produced and attached with male gametes during the induction process. Afterwards they doubled to develop into diploid in the subsequent process. At the same time, there was a certain degree of specific insertion and paternal genome loss. Our study establishes an understanding toward the mechanism of DH induction and its findings can be used in DH breeding to save time and money.

## Methods

### Plant materials

The rapeseed genotypes used in this experiment are shown in Table 3. These genotypes include allo-octoploid (2n = 8× ≈ 76, AAAACCCC) rapeseed: double haploid induction lines Y3380, Y3560, HZ23 and HZ28, bred by Chengdu Academy of Agriculture and Forestry Sciences. HZ23 is a selfed progeny of Y3560, and HZ28 is the double F_1_ generation of hybrid *Brassica napus* 908 (2n = 8× ≈ 76). 3380 and 3560 (3560–1 ~ 3560–5, derived from the segregating progeny of Y3560) are mixed ploidy. 3850 (2n = 6× ≈ 58, AAAACC), HZ1 (2n = 6× = 54, AABBCC), HZ2 (2n = 6× = 54, BBCCAA) and HZ4 (2n = 6× ≈ 58, AAAACC) are hexaploidy rapeseed. Among these genotypes, 3850 is the self-segregated offspring of Y3380, while HZ4 is an artificially doubled F_1_ offspring of B0486 (2n = 4× = 38, AACC, *Brassica napus*) × Ya’an Huang (YH, 2n = 2× = 20), provided by Chengdu Academy of Agriculture and Forestry Sciences. HZ1 (AABBCC) and HZ2 (BBCCAA) were provided by Huazhong Agricultural University. HZ24, 3114, 3128, DW39, 3560–6, P3–2, 3737, 4417 and W1A were tetraploid *Brassica napus*. HZ24 (2n = 4× = 38, AACC), common *Brassica napus* 3114 (2n = 4× = 38, AACC) and 3128 (2n = 4× = 38, AACC) were self-segregated progeny of Y3380 *Brassica napus* (2n = 4× = 38, AACC). DW39 and 3560–6 (2n = 4× = 38, AACC) were self-segregated offspring of Y3560. P3–2 (AACC, 2n = 4× =38) is the main parent material created by Y3380 and Y3560. Three thousand seven hundred thirty-seven is a common *Brassica napus* (2n = 4× = 38, AACC) and 4417 (2n = 4× = 38, AACC) is a Y3380 × Y3560 segregated tetraploid offspring; WlA (2n = 4× = 38, AACC) is a wax-less *polima* cytoplasmic sterile line, used as an induction or hybrid tester species. At the same time, the wax-less trait is controlled by a single gene [56]. T194 (2n = 3× = 29) is a triploid rapeseed (F_1_ generation) synthesized between species [P3–2 × HZ32 (2n = 2× = 20, *Brassica rape*)] and it was used as the rapeseed DH induction line. T195 (2n = 3× = 29) is also a triploid rapeseed (F_1_ generation) which is interspecies of ZS11 (2n = 4× = 38, *Brassica napus*) × HZ32 (2n = 2× = 20, *Brassica rape*) and has no role in DH induction lines. See Fig. 5 for some sources of materials related to DH induction lines. All plant materials were planted in Wenjiang experimental base of Chengdu Academy of Agriculture and Forestry Sciences (E103.83, N30.70). The collection and breeding of the *B. napus* materials were obtained and used with local permission of China Germplasm Regulation Authorities. The *B.napus* used in this study was commonly grown as oil crop and considered as native species of China and does not fall under the Nagoya protocol.

**Table 3:** Material information table

Material name	Ploidy	Source of material	Remarks	Wax or waxless
Y3380	Octoploid (2n = 8× ≈ 76, AAAACCCC)	Provided by Chengdu Academy of Agriculture and Forestry Sciences	Related to DH induction line	wax
Y3560				
HZ23				
HZ28				
3380–1	Mixed ploidy (2n = 4\6\8×)			
3560–1 ~ 3560–5				
3850	Hexaploid (2n = 6× ≈ 58, AAAACC)			
3114	Tetraploid (2n = 4× = 38, AACC)			
3128				
DW39				
3560–6				
P3–2				
4417				
T194	Triploid (2n = 3× ≈ 29, AAC)			
HZ1	Hexaploid (2n = 6× ≈ 57, AABBCC)	Provided by Huazhong Agricultural University	Not related to DH induction line	
HZ2				
HZ4				
HZ24	Tetraploid (2n = 4× ≈ 38, AACC)	Provided by Chengdu Academy of Agriculture and Forestry Sciences		
3737				
T195	Triploid (2n = 3× ≈ 29, AAC)			
WlA	*pol* Cytoplasmic sterile line, Tetraploid (2n = 4× = 38, AACC)			waxless

The materials include octoploid rapeseed: doubled haploid induction lines Y3380 (2n = 8× ≈ 76, AAAACCCC) and Y3560 (2n = 8× ≈ 76, AAAACCCC), HZ23 (2n = 8× ≈ 76, AAAACCCC) and HZ28 (2n = 8× ≈ 76, AAAACCCC). HZ23 is the selfed progeny of Y3560, and HZ28 is the doubled F_1_ generation of hybrid *Brassica napus* 908 (2n = 8× ≈ 76). Mixed ploidy: 3380 and 3560 (3560–1 ~ 3560–5, derived from the self-separating progeny of Y3560). Hexaploid rapeseed: 3850 (2n = 6× ≈ 58, AAAACC), HZ1 (2n = 6× = 54, AABBCC), HZ2 (2n = 6× = 54, BBCCAA) and HZ4 (2n = 6× ≈ 58, AAAACC). Among them, 3850 is the self-separated offspring of Y3380, and HZ4 is the artificially doubled offspring of B0486 (2n = 4× = 38, AACC, *Brassica napus*) × Ya’an Huang (YH, 2n = 2× = 20)) F_1_ generation. HZ1 (AABBCC) and HZ2 (BBCCAA) are provided by Huazhong Agricultural University. Tetraploid *Brassica napus*: HZ24, 3114, 3128, DW39, 3560–6, P3–2, 3737, 4417 and W1A. HZ24 (2n = 4× = 38, AACC) ordinary *Brassica napus*; 3114 (2n = 4× = 38, AACC) and 3128 (2n = 4× = 38, AACC) are Y3380 self-separated *Brassica napus* (2n = 4× = 38, AACC); DW39 and 3560–6 (2n = 4× = 38, AACC) are self-separating offspring of Y3560; P3–2 (AACC, 2n = 4 × = 38) is the core parent material created by Y3380 and Y3560; 3737 is a common *Brassica napus* (2n = 4× = 38, AACC); 4417 (2n = 4× = 38, AACC) is a Y3380 × Y3560 separated tetraploid offspring; WlA (2n = 4× = 38, AACC) is a waxless *polima* cytoplasmic sterile line, used as an induction or hybrid tester species. T194 (2n = 3× = 29) is a triploid rapeseed (F_1_ generation) synthesized between species (P3–2 (2n = 4× = 38, *Brassica napus*) × HZ32 (2n = 2× = 20, *Brassica rape*)) and related to the rapeseed DH induction line. T195 (2n = 3× = 29) is a triploid rape (F_1_ generation) that is interspecies (ZS11 (2n = 4× = 38, *Brassica napus*) × HZ32 (2n = 2× = 20, *Brassica rape*)) and has nothing to do with the DH induction lines

### Material pollination

Uniformly growing and healthy wax-less (WlA) plants were selected for pollination, that were bagged before flowering. Male parent plants were also bagged before blooming to prevent the loss of pollen powder. Later on, the pollens of male parents were used to pollinate the wax-less (WlA) *Brassica napus* plants and covered again. The hybridization combinations derived from the cross of WlA and other male parental materials are shown in Additional file 15.

### Phenotypic identification

We identify and counted the number of plants, with and without wax powder in the F_1_ generation of the hybrid combination between 10 and 11 am on the basis of their phenotypic appearance. Representative plants are selected and photographed with a SLR camera (E0S 200D, Canon).

### Identification of ploidy via flow cytometry

Flow cytometry has been used to assess plant ploidy [57]. In this research, flow cytometry is used to identify ploidy in all parental and F_1_ hybrid combinations [58]. We took the fresh young leaves between 9 and 11 am, washed them with distilled water, and wiped off the surface debris with a filter paper or a punch. A leaf with a diameter of 0.5 cm were selected and placed in a pre-cooled petri dish. Subsequently, added 0.5 ml of pre-chilled LB01 cell lysis buffer (15 mM Tris, 2 mM disodium edetate, 0.5 mM spermine tetrahydrochloride, 80 mM potassium chloride, 20 mM sodium chloride, 0.1% Triton-100 and 15 mM β-mercaptoethanol, pH 7.5, filtered with 0.22 mm filter membrane). The leaves were quickly cut into pieces with a blade and filtered through a 35 mm filter into a 2 ml EP tube. After that 1 ml PI staining solution (5% propidium iodide and 5% RNase) was added in the dark for 30 min, and then flowed load on the cytometer (Accuri C6 Plus, BD). At least 10,000 cells were collected in one sample and the data were analyzed using AccuriC software.

### Observation of meiosis

The observation of meiotic behavior was carried out according to the previously described method of Li [59]. To study the behavior of chromosomes, the young flower buds with the cleavage phase of pollen mother cells (PMCs) were placed in Carnoy’s fixative (ethanol: glacial acetic acid, 3:1, v/v) for 24 h. Small anthers were taken and dissociated in 1 M hydrochloric acid at 60 °C for 6–8 min, and place them on a glass slide to gently release the pollen mother cells and spores, thenceforth dropped the carbo fuchsin solution and observed under an optical microscope.

### Count of somatic chromosome

Young flower buds were used for counting of somatic chromosomes. Flower buds were collected at 9 am, treated them with 0.002 mol/L 8-hydroxyquinoline solution and placed them in the dark for 3 h, and then transferred to Carnot fixative (ethanol: glacial acetic acid, 3: 1, v/v) to fix 24 h. The number of somatic chromosomes was identified by microscopic observation of somatic cells. The cytogenetic observation procedure was followed as described in a previous study [60].

### SNP chip scanning

On the basis of phenotype identification and flow cytometry ploidy results, the parents and hybrid combination samples of F_1_ generation were selected (3 individual plants with and without wax powder). The source of SNP test samples is shown in Additional file 16. 50 K SNP chip (the chip is made by Illumina used in current experiment and were tested and analyzed by Wuhan Shuanglvyuan Chuangxin Technology Research Institute Co., Ltd. (Additional file 17). Noviza FastPure Plant DNA Isolation Mini Kit-DC104 was used to extract DNA from young leaves, according to DNA concentration > 50 ng/ul, total DNA > 1.75μg, and checked its absorbance value A260/A280 between 1.8 ~ 2.0. All the screen samples qualified the to the standards given by manufacturer.

### Analysis of homozygous site rate in sample SNP

The GType format of Illumina chips ultimately has four situations: AA, BB, AB, and NC. Using the effective sites detected by the 50 K SNP chip, we used the following formula to calculate the rate of SNP homozygous sites.

$$\mathsf{SNP}\ \mathsf{homozygous}\ \mathsf{site}\ \mathsf{rate}=\left(\mathsf{AA}+\mathsf{BB}\right)\ \mathsf{SNP}\ \mathsf{site}\ \mathsf{number}/\mathsf{SNP}\ \mathsf{effective}\ \mathsf{site}\ \mathsf{number}$$

### Analysis of plant genotyping

We used a self-developed Perl script to screen out the homozygous and heterozygous sites of the same parental site, then compared the same and hybridization sites between the offspring and the parents and counted the number of sites. Afterwards we calculated the probability and draw the genotyping map (Additional file 14).

### Sample cluster analysis

By comparing the position differences between the two samples, an n x n-dimensional matrix was obtained. The apply function of R language counts the SNP difference between each sample and other samples to form a distance matrix (Additional file 18), and later on hclust function was used to perform hierarchical clustering [61].

## Supplementary Information

**Additional file 1.** G1 phase flow cytometric fluorescence value of parents.

**Additional file 2.** Chromosome number of triploid sample and flow cytometry result. a Flow cytometry histogram of T194. b Chromosome number of T194, 2n = 29. c Flow cytometry histogram of T195. d Chromosome number of T195, 2n = 29. Scale bar:10 μm.

**Additional file 3.** The number of chromosomes of the tetraploid sample and the flow cytometry result. a Flow cytometry histogram of 3114. b Chromosome number of 3114, 2n = 38. c Flow cytometry histogram of 3128. d Chromosome number of 3128, 2n = 38. e Flow cytometry histogram of 3560–6. f Chromosome number of 3560–6, 2n = 38. g Flow cytometry histogram of DW39. h Chromosome number of DW39, 2n = 38. i Flow cytometry histogram of P3–2. j Chromosome number of P3–2, 2n = 38. k Flow cytometry histogram of 3737. l Chromosome number of 3737, 2n = 38. m Flow cytometry histogram of HZ24. n Chromosome number of HZ24, 2n = 38. Scale bar:10 μm.

**Additional file 4.** The number of chromosomes in the hexaploid sample and the flow cytometry results. a Flow cytometry diagram of 3850. b Chromosome number of 3850, 2n = 54. c Flow cytometry histogram of HZ1. d Chromosome number of HZ1. e Flow cytometry histogram of HZ4. f Chromosome number of HZ4, 2n = 52. Scale bar:10 μm.

**Additional file 5.** The number of chromosomes of octoploid samples and the results of flow cytometry. a Flow cytometry histogram of 3560–1. b-d Chromosome number of 3560–1(2n = 36, 2n = 74, 2n = 74). e Flow cytometry histogram of Y3560. f Chromosome number of Y3560, 2n = 66. g Flow cytometry histogram of HZ23. h Chromosome number of HZ23, 2n = 70. i Flow cytometry histogram of HZ28. j Chromosome number of HZ28. Scale bar:10 μm.

**Additional file 6.** The meiotic process of pollen mother cell of triploid material T194. a Meiosis prophase I. b Meiosis prophase I. c Meiosis prophase I. d Meiosis anaphase I. Scale bar:10 μm.

**Additional file 7.** Summary of F_1_ generation flow cytometry fluorescence value and phenotype identification.

**Additional file 8.** Partial F_1_ generation chromosome observation. a Female parent (WlA), 2n = 38. b Haploid F_1_ generation of WlA × 3114, 2n = 19. c Hybrid tetraploid F_1_ generation of WlA × 3114, 2n = 38. d Induced F_1_ generation of WlA × 3850, 2n = 38. e Hybrid F_1_ generation of WlA × HZ23, 2n = 62. f Induced F_1_ generation of WlA × 3560–1, 2n = 38. Scale bar:10 μm.

**Additional file 9.** Chromosome number identification part of F_1_ generation flow cytometry results.

**Additional file 10.** SNP density distribution map on the chromosome.

**Additional file 11.** Genetic cluster analysis of parents and F_1_ generation.

**Additional file 12.** Variance analysis of induction rate. It means that there is a significant difference between the induction rate of 3737 and the other 7 materials (P3–2, 3560–6, HZ28, 3114, 3128, 4417 and DW39), ns means the difference is not significant.

**Additional file 13.** Abnormal behavior of P3–2 chromosome. a Mitosis. b Mitosis. c The tetrad period of meiosis. The arrow in the figure points to a lagging chromosome. Scale bar:10 μm.

**Additional file 14.** Summary of Genotyping results.

**Additional file 15.** Cross combination table.

**Additional file 16.** SNP test sample list.

**Additional file 17.** 50 K SNP chip raw data. a Maternal parent (WlA), tetraploid male parent material (3560–6, P3–2, HZ24, 3114, 3128, 4417, 3737, and DW39) and its genotyping classification map of F_1_ generation. b Maternal parent (WlA), octoploid male parent material (Y3560, Y3380, HZ23 and HZ28) and its genotyping classification map of F_1_ generation. c Maternal parent (WlA), triploid male parent material (T194, T195) and genotyping classification chart of F_1_ generation. d Maternal parent (WlA), hexaploid male parent material (3850, HZ1 and HZ4) and its genotyping classification map of F_1_ generation. e Maternal parent (WlA), mixed male parent materials (3380–1, 3560–1 and 3560–2) and their genotyping classification maps of F_1_ generation. The maternal (W1A) locus is shown in gray, the paternal locus is shown in red, and the hybridization site is shown in blue.

**Additional file 18.** Genetic distance matrix.

## Data Availability

The datasets supporting the conclusions of this article are included within the article and its additional files.
