# Supplementary figures and images for: Genetic characteristics and ploidy trigger the high inducibility of double haploid (DH) inducer in Brassica napus
Source: BMC Plant Biol. 2021 Nov 16;21:538. doi: 10.1186/s12870-021-03311-z (PMC8594162; doi:10.1186/s12870-021-03311-z)

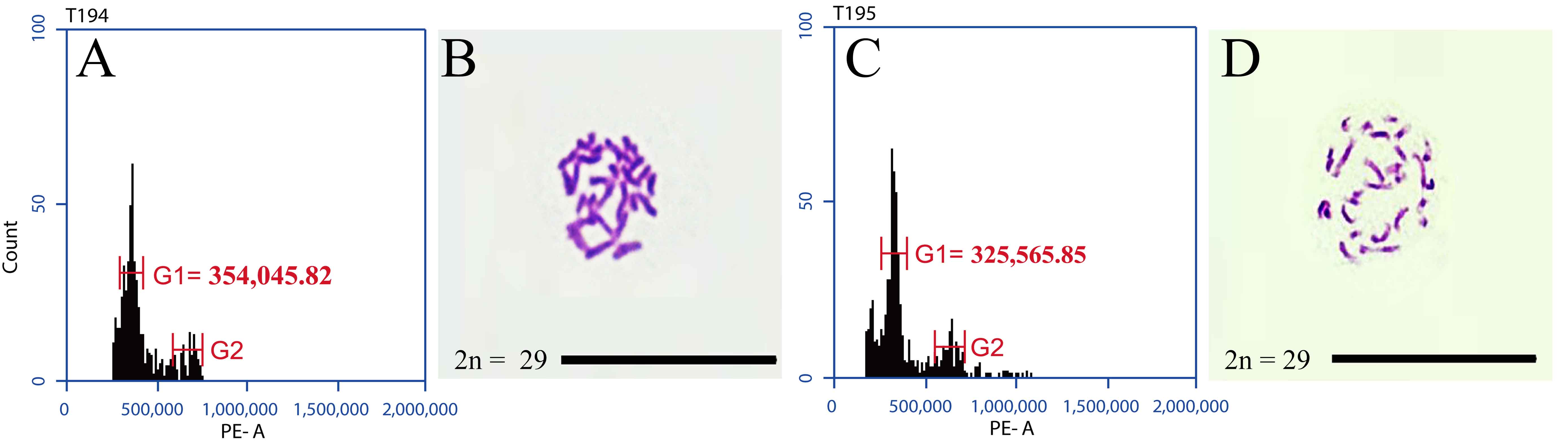

Supplement: Supplementary file 2 — Additional file 2. Chromosome number of triploid sample and flow cytometry result. a Flow cytometry histogram of T194. b Chromosome number of T194, 2n = 29. c Flow cytometry histogram of T195. d Chromosome number of T195, 2n = 29. Scale bar:10 μm. [file 12870_2021_3311_MOESM2_ESM.jpg]

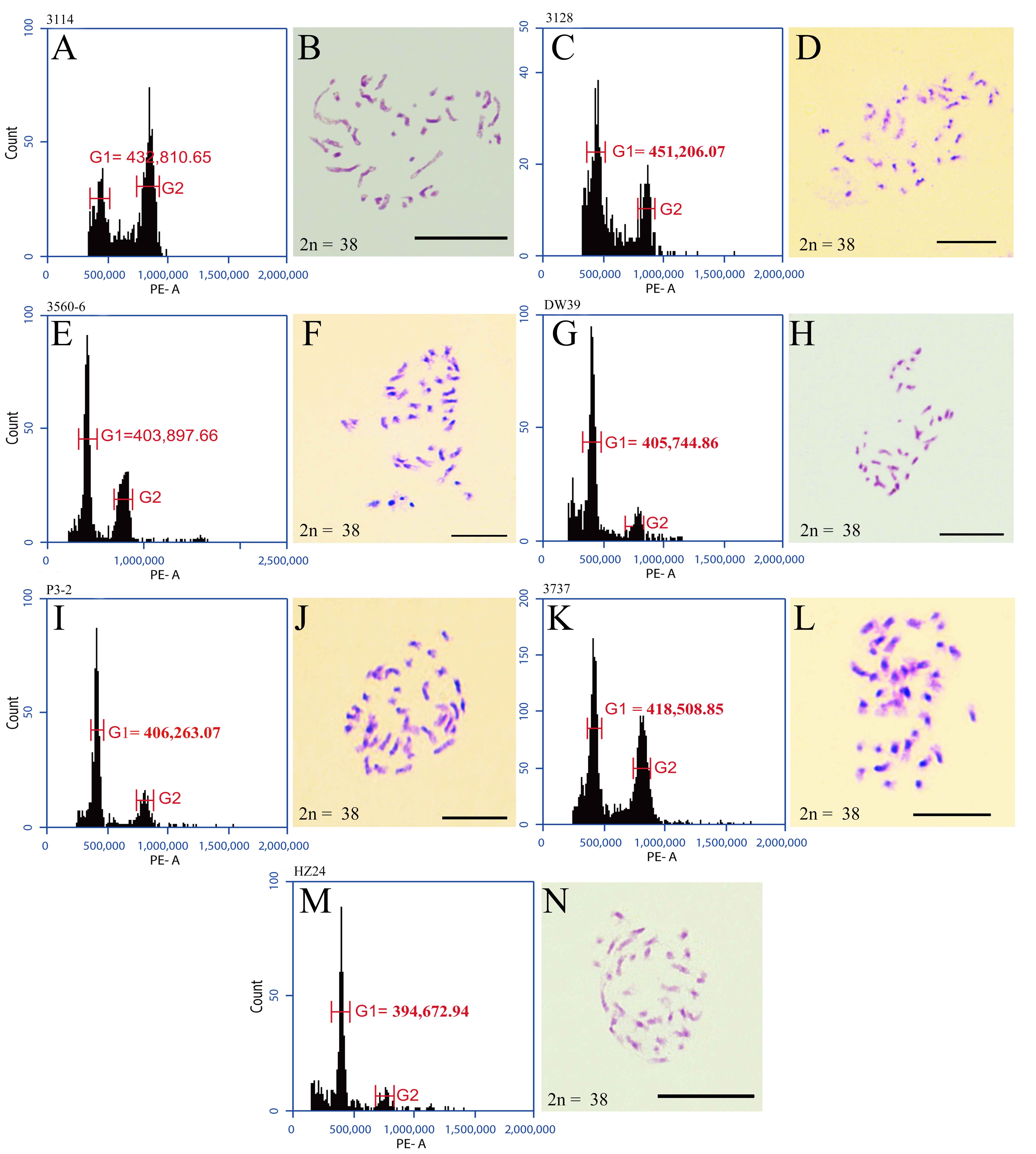

Supplement: Supplementary file 3 — Additional file 3. The number of chromosomes of the tetraploid sample and the flow cytometry result. a Flow cytometry histogram of 3114. b Chromosome number of 3114, 2n = 38. c Flow cytometry histogram of 3128. d Chromosome number of 3128, 2n = 38. e Flow cytometry histogram of 3560–6. f Chromosome number of 3560–6, 2n = 38. g Flow cytometry histogram of DW39. h Chromosome number of DW39, 2n = 38. i Flow cytometry histogram of P3–2. j Chromosome number of P3–2, 2n = 38. k Flow cytometry histogram of 3737. l Chromosome number of 3737, 2n = 38. m Flow cytometry histogram of HZ24. n Chromosome number of HZ24, 2n = 38. Scale bar:10 μm. [file 12870_2021_3311_MOESM3_ESM.jpg]

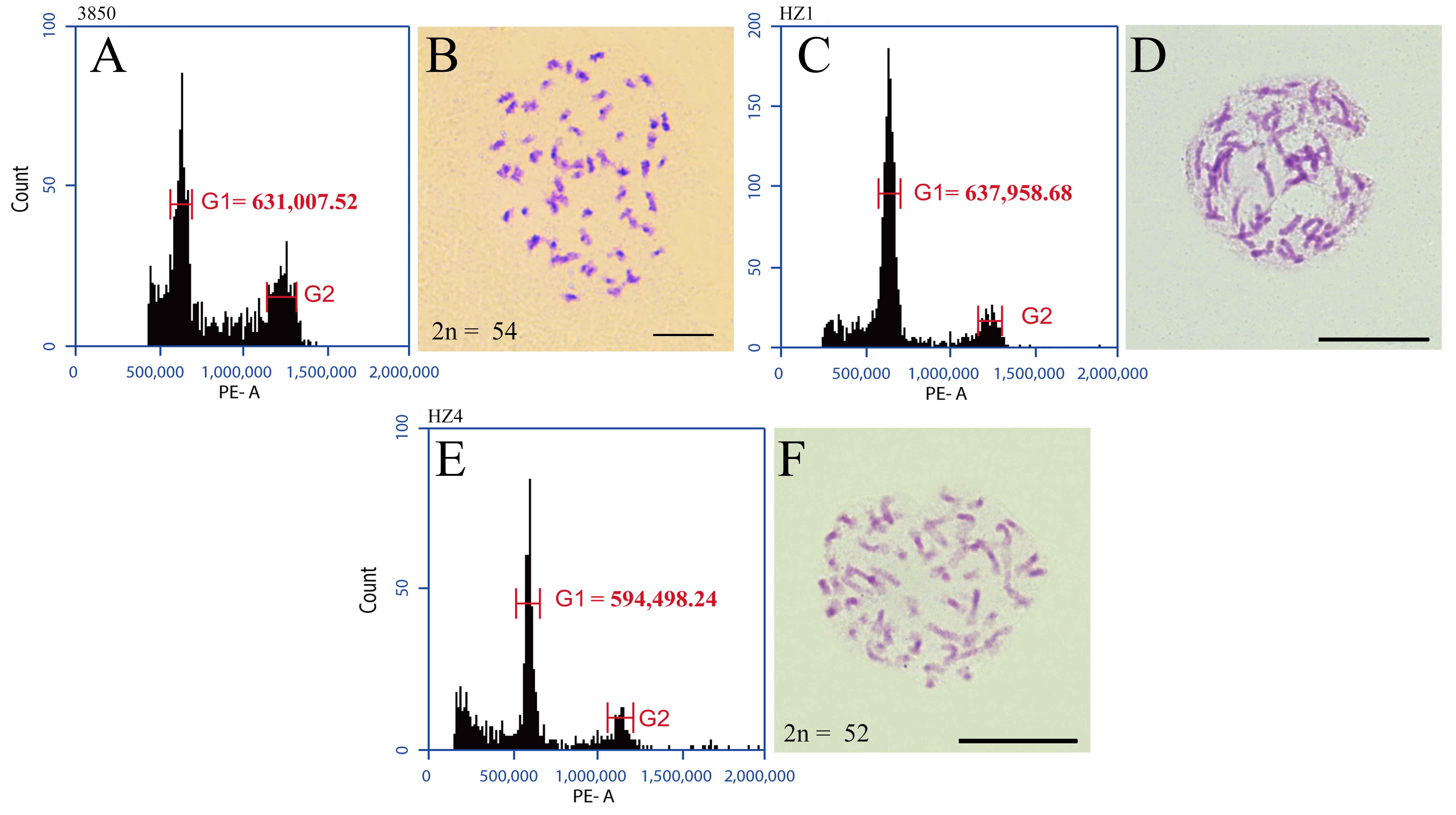

Supplement: Supplementary file 4 — Additional file 4. The number of chromosomes in the hexaploid sample and the flow cytometry results. a Flow cytometry diagram of 3850. b Chromosome number of 3850, 2n = 54. c Flow cytometry histogram of HZ1. d Chromosome number of HZ1. e Flow cytometry histogram of HZ4. f Chromosome number of HZ4, 2n = 52. Scale bar:10 μm. [file 12870_2021_3311_MOESM4_ESM.jpg]

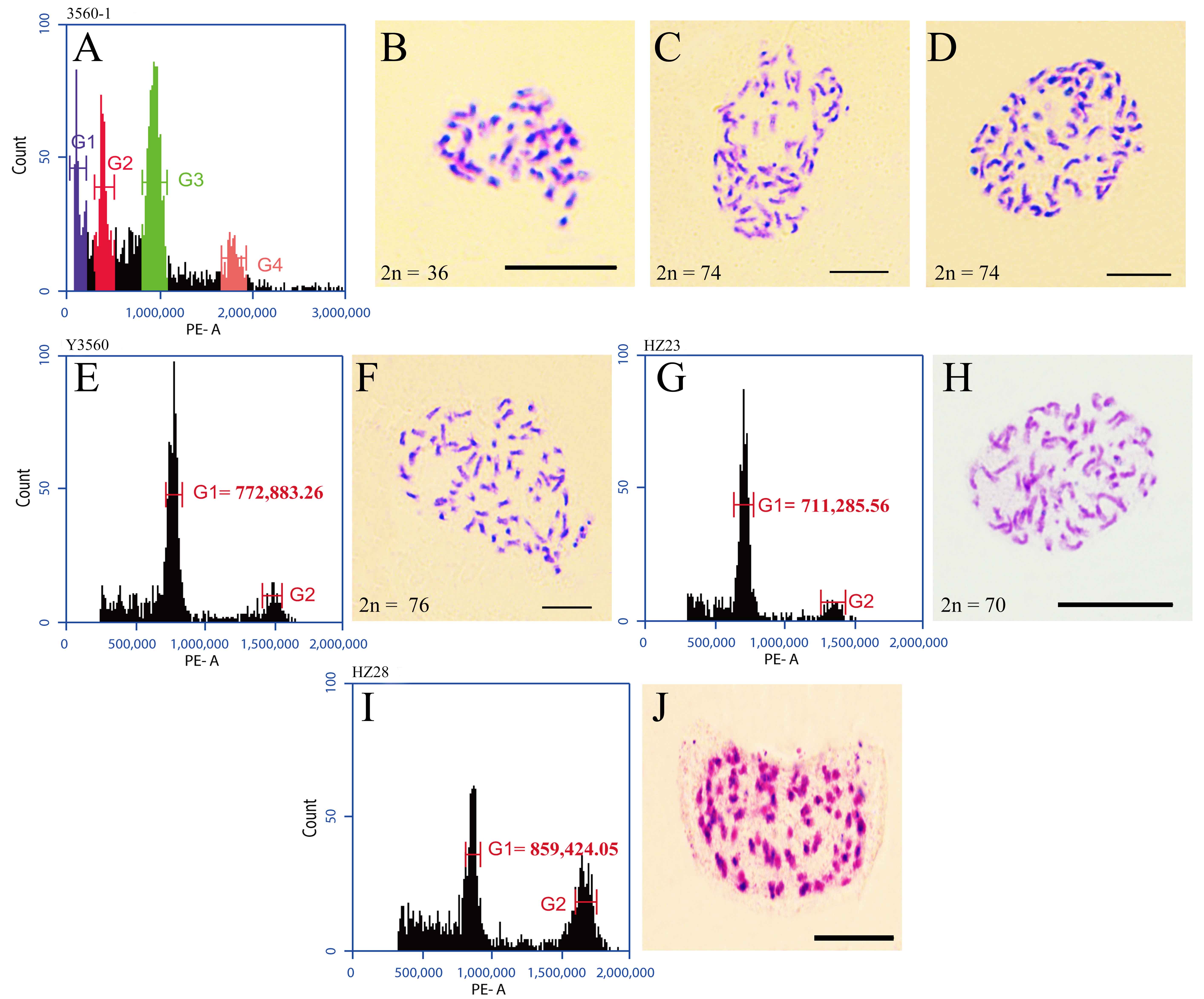

Supplement: Supplementary file 5 — Additional file 5. The number of chromosomes of octoploid samples and the results of flow cytometry. a Flow cytometry histogram of 3560–1. b-d Chromosome number of 3560–1(2n = 36, 2n = 74, 2n = 74). e Flow cytometry histogram of Y3560. f Chromosome number of Y3560, 2n = 66. g Flow cytometry histogram of HZ23. h Chromosome number of HZ23, 2n = 70. i Flow cytometry histogram of HZ28. j Chromosome number of HZ28. Scale bar:10 μm. [file 12870_2021_3311_MOESM5_ESM.jpg]

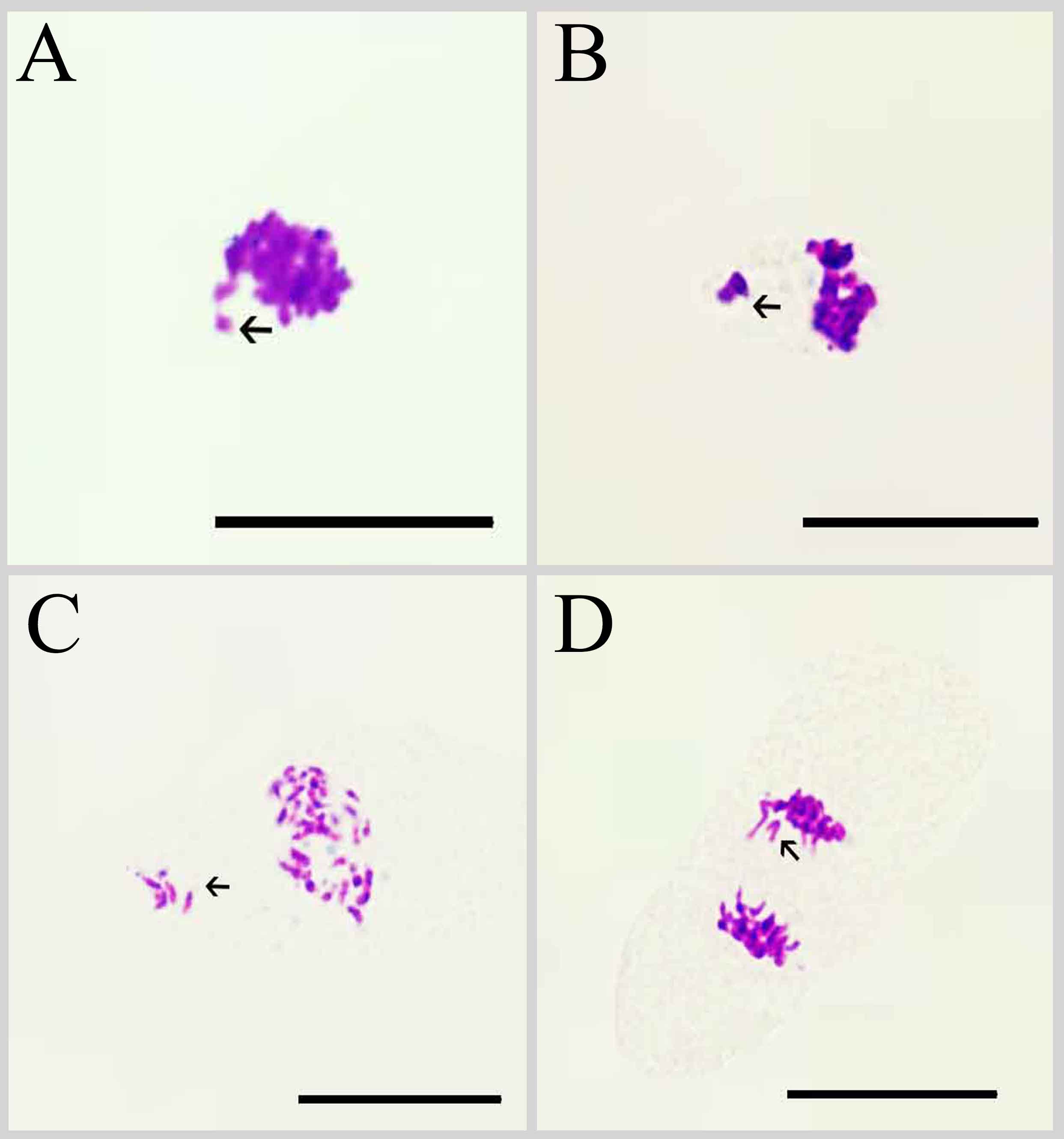

Supplement: Supplementary file 6 — Additional file 6. The meiotic process of pollen mother cell of triploid material T194. a Meiosis prophase I. b Meiosis prophase I. c Meiosis prophase I. d Meiosis anaphase I. Scale bar:10 μm. [file 12870_2021_3311_MOESM6_ESM.jpg]

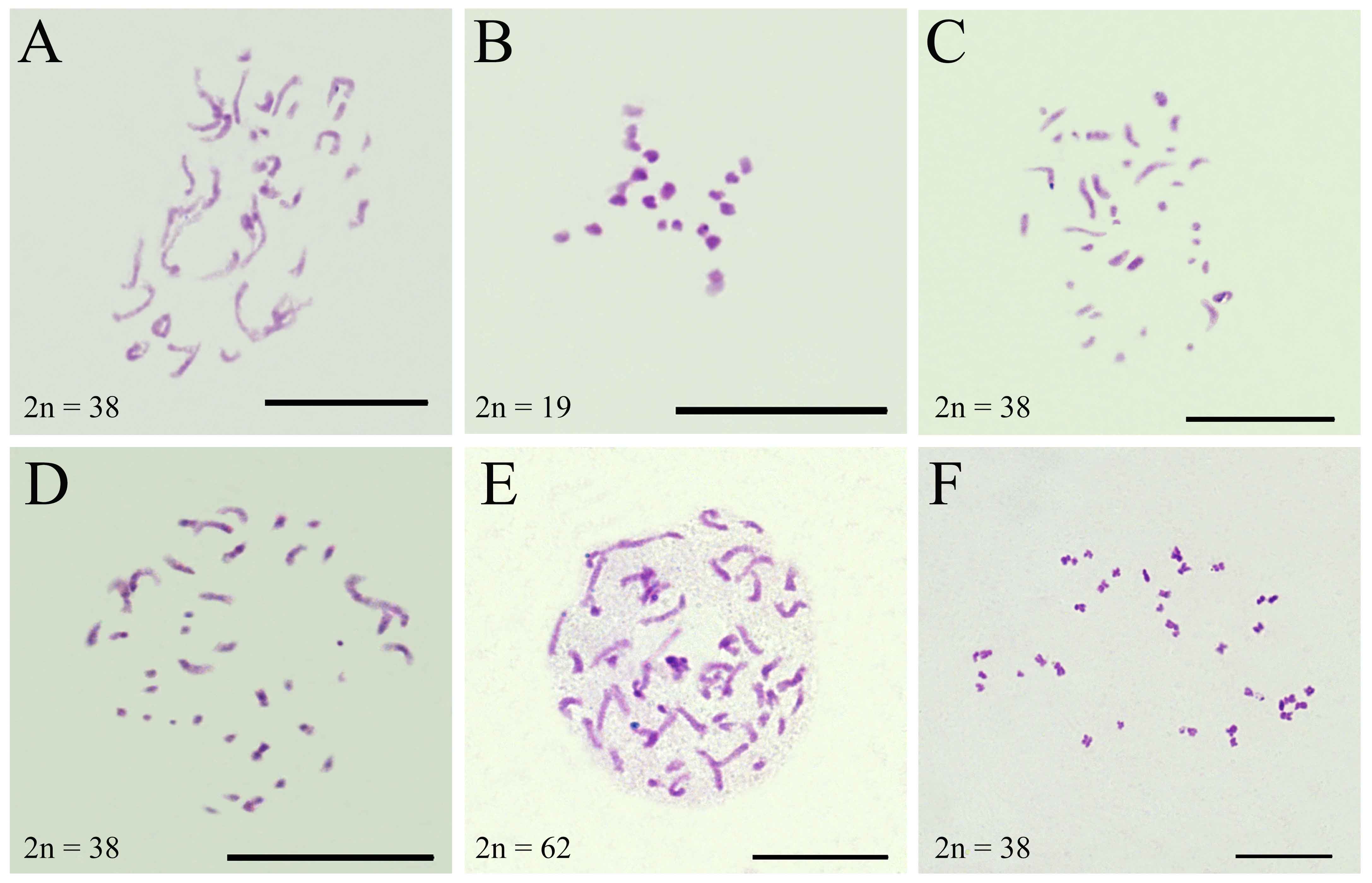

Supplement: Supplementary file 8 — Additional file 8. Partial F1 generation chromosome observation. a Female parent (WlA), 2n = 38. b Haploid F1 generation of WlA × 3114, 2n = 19. c Hybrid tetraploid F1 generation of WlA × 3114, 2n = 38. d Induced F1 generation of WlA × 3850, 2n = 38. e Hybrid F1 generation of WlA × HZ23, 2n = 62. f Induced F1 generation of WlA × 3560–1, 2n = 38. Scale bar:10 μm. [file 12870_2021_3311_MOESM8_ESM.jpg]

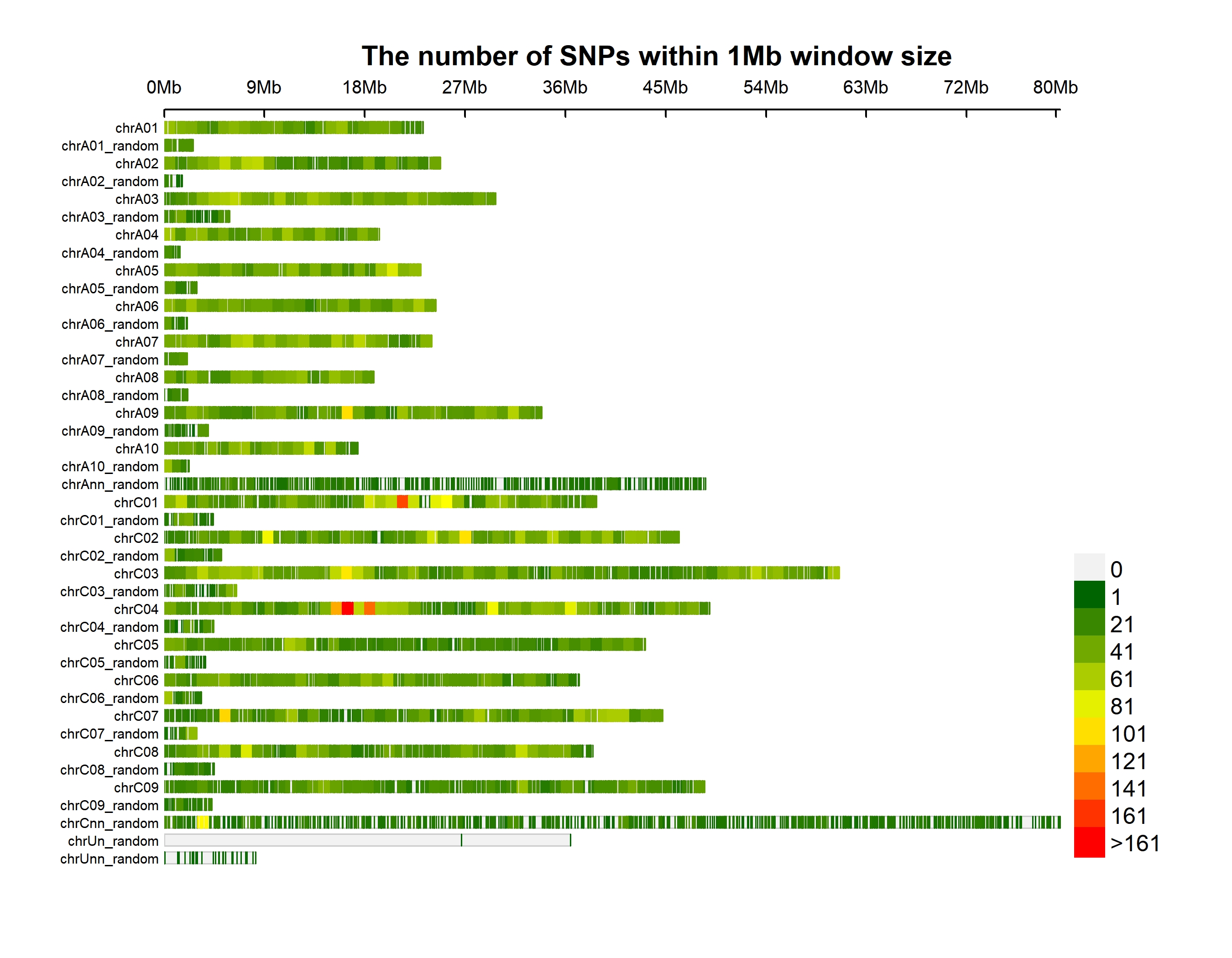

Supplement: Supplementary file 10 — Additional file 10. SNP density distribution map on the chromosome. [file 12870_2021_3311_MOESM10_ESM.jpg]

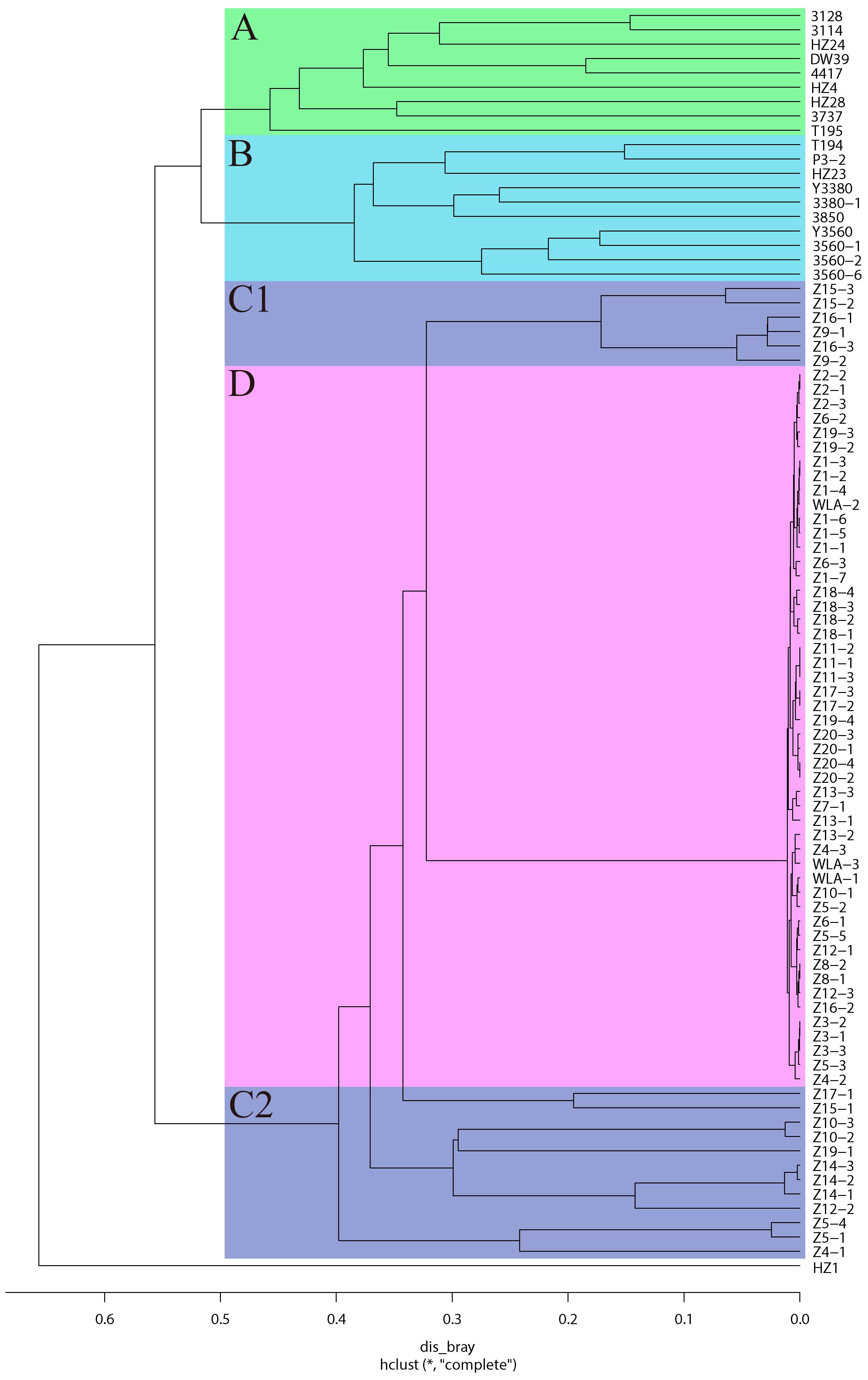

Supplement: Supplementary file 11 — Additional file 11. Genetic cluster analysis of parents and F1 generation. [file 12870_2021_3311_MOESM11_ESM.jpg]

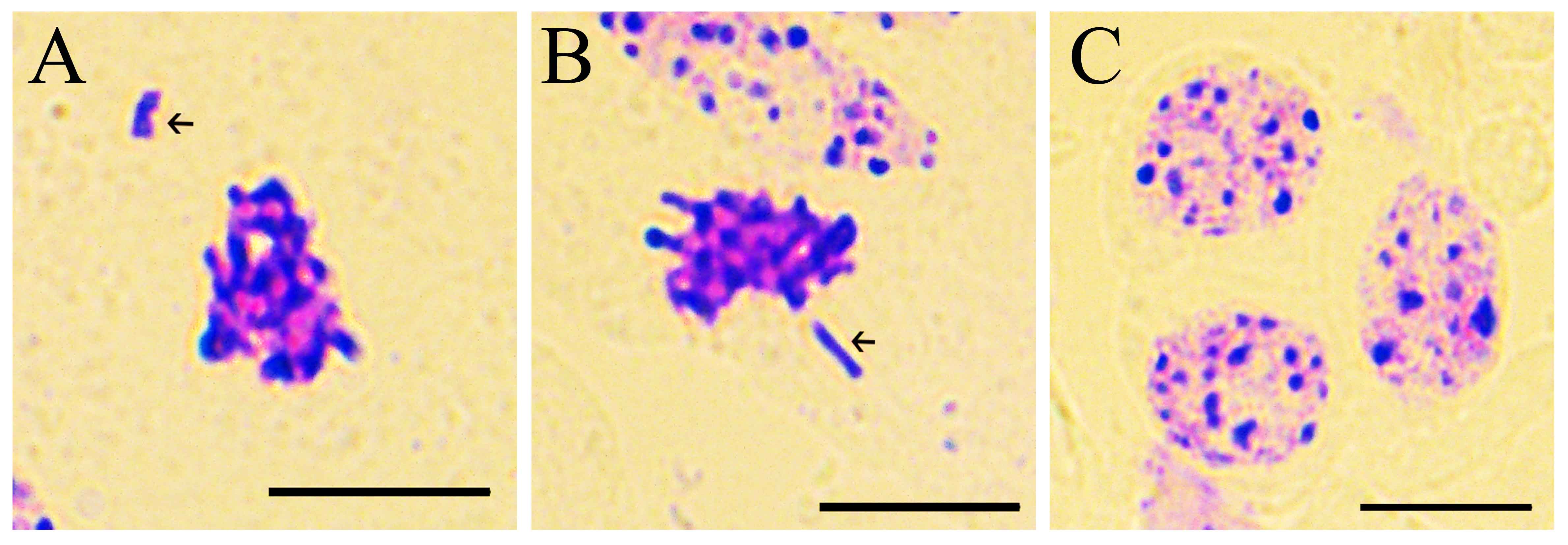

Supplement: Supplementary file 13 — Additional file 13. Abnormal behavior of P3–2 chromosome. a Mitosis. b Mitosis. c The tetrad period of meiosis. The arrow in the figure points to a lagging chromosome. Scale bar:10 μm. [file 12870_2021_3311_MOESM13_ESM.jpg]

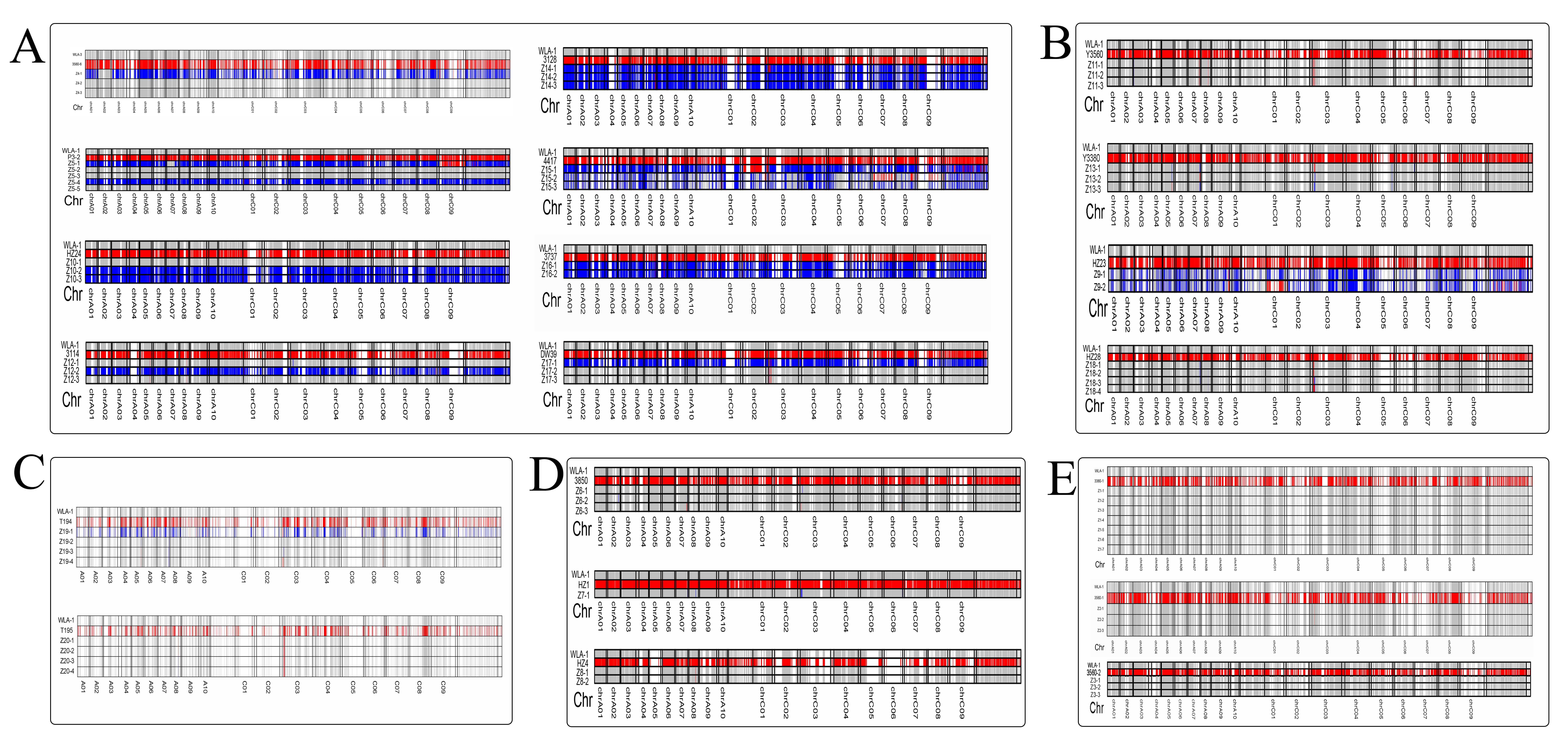

Supplement: Supplementary file 14 — Additional file 14. Summary of Genotyping results. [file 12870_2021_3311_MOESM14_ESM.jpg]
